# The complex polyploid genome architecture of sugarcane

**DOI:** 10.1038/s41586-024-07231-4

**Published:** 2024-03-27

**Authors:** A. L. Healey, O. Garsmeur, J. T. Lovell, S. Shengquiang, A. Sreedasyam, J. Jenkins, C. B. Plott, N. Piperidis, N. Pompidor, V. Llaca, C. J. Metcalfe, J. Doležel, P. Cápal, J. W. Carlson, J. Y. Hoarau, C. Hervouet, C. Zini, A. Dievart, A. Lipzen, M. Williams, L. B. Boston, J. Webber, K. Keymanesh, S. Tejomurthula, S. Rajasekar, R. Suchecki, A. Furtado, G. May, P. Parakkal, B. A. Simmons, K. Barry, R. J. Henry, J. Grimwood, K. S. Aitken, J. Schmutz, A. D’Hont

**Affiliations:** 1 Genome Sequencing Center, HudsonAlpha Institute for Biotechnology, Huntsville, AL USA; 2 CIRAD, UMR AGAP Institut, Montpellier, France; 3 UMR AGAP Institut, Univ Montpellier, CIRAD, INRAE, Institut Agro, Montpellier, France; 4 Department of Energy Joint Genome Institute, Lawrence Berkeley National Laboratory, Berkeley, CA USA; 5 Sugar Research Australia, Te Kowai, Queensland Australia; 6 Corteva Agriscience, Johnston, IA USA; 7 CSIRO Agriculture and Food, Queensland Bioscience Precinct, St Lucia, Queensland Australia; 8 Institute of Experimental Botany of the Czech Academy of Sciences, Centre of Plant Structural and Functional Genomics, Olomouc, Czech Republic; 9 ERCANE, Sainte-Clotilde, La Réunion, France; 10 Arizona Genomics Institute, University of Arizona, Tucson, AZ USA; 11 CSIRO Agriculture and Food, Urrbrae, South Australia Australia; 12 Queensland Alliance for Agriculture and Food Innovation, University of Queensland, Brisbane, Queensland Australia; 13 Joint BioEnergy Institute, Lawrence Berkeley National Laboratory, Emeryville, CA USA; 14 ARC Centre of Excellence for Plant Success in Nature and Agriculture, University of Queensland, Brisbane, Queensland Australia

**Keywords:** Polyploidy in plants, Genome informatics, Plant genetics, Agriculture, Genome evolution

## Abstract

Sugarcane, the world’s most harvested crop by tonnage, has shaped global history, trade and geopolitics, and is currently responsible for 80% of sugar production worldwide^1^. While traditional sugarcane breeding methods have effectively generated cultivars adapted to new environments and pathogens, sugar yield improvements have recently plateaued^2^. The cessation of yield gains may be due to limited genetic diversity within breeding populations, long breeding cycles and the complexity of its genome, the latter preventing breeders from taking advantage of the recent explosion of whole-genome sequencing that has benefited many other crops. Thus, modern sugarcane hybrids are the last remaining major crop without a reference-quality genome. Here we take a major step towards advancing sugarcane biotechnology by generating a polyploid reference genome for R570, a typical modern cultivar derived from interspecific hybridization between the domesticated species (*Saccharum officinarum*) and the wild species (*Saccharum spontaneum*). In contrast to the existing single haplotype (‘monoploid’) representation of R570, our 8.7 billion base assembly contains a complete representation of unique DNA sequences across the approximately 12 chromosome copies in this polyploid genome. Using this highly contiguous genome assembly, we filled a previously unsized gap within an R570 physical genetic map to describe the likely causal genes underlying the single-copy *Bru1* brown rust resistance locus. This polyploid genome assembly with fine-grain descriptions of genome architecture and molecular targets for biotechnology will help accelerate molecular and transgenic breeding and adaptation of sugarcane to future environmental conditions.

## Abstract (web summary)

We build a polyploid reference genome for hybrid sugarcane cultivar R570, improving on its current ‘mosaic monoploid’ representation, enabling fine-grain description of genome architecture and the exploration of candidate genes underlying the *Bru1* brown rust resistance locus.

## Main

Sugarcane domestication began approximately 10,000 years ago with the first ‘sweet’ cultivars (*Saccharum officinarum*) derived from *Saccharum robustum*^3^. Modern day cultivars, however, are all derived from a few interspecific hybridizations performed by breeders a century ago between ‘sweet’ octoploid *S. officinarum* and the ‘wild’ polyploid *Saccharum spontaneum*. Sugarcane interspecific hybridization has provided major breakthroughs in disease resistance and adaptation to otherwise stressful environmental conditions. However, early generation hybrids also had much lower sugar yield, owing to the large wild genomic contribution. To re-establish high sugar yield, breeders backcrossed hybrids to *S. officinarum*^4^. This process was accelerated by the unreduced (‘2*n*’) transmission of *S. officinarum* chromosomes in the first two generations so backcrossed (BC1) cultivars contained 11% more domesticated sequence than would be expected by typical (*n* + *n*) inheritance patterns.

While interspecific hybridization and backcrossing represent crucial steps for modern sugarcane breeding, they produced cultivars with extraordinarily complex genomes. In addition to variable progenitor subgenome dosage (due to unreduced ‘2*n*’ gamete transmission), hybrid sugarcane meiotic recombination and chromosome pairing is variable within and among progenitor subgenomes. Chromosome pairing is mainly bivalent (although meiotic abnormalities can occur)^5–7^ but with differential pairing affinity between chromosomes, leading to a continuum of polysomic inheritance (with random association between homologues) and disomic inheritance (with systematic association between a pair of homologues)^8–10^. Recombination between progenitor subgenomes can also generate ‘interspecific recombinant’ chromosomes that contain both ‘wild’ and ‘sweet’ ancestry. As a result, chromosomes may be highly heterozygous, translocated, inherited purely from progenitor genomes, aneuploid, interspecific recombinant or entirely identical-by-descent to another chromosome. These processes result in a diverse and complex hybrid sugarcane genome.

## The road to a representative genome

**Fig. 1:**
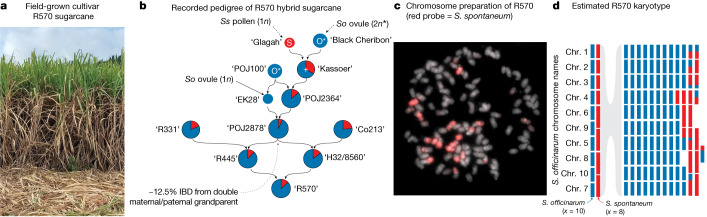
The pedigree and genome organization of R570 hybrid sugarcane. **a**, An image of field-grown R570 (approximately 4 m in height). **b**, Estimated recorded pedigree of the R570 in **a**. Standardized contributions of progenitor genomes (red, *S. spontaneum* (*Ss*), ‘wild‘ sugarcane; blue, ‘sweet’ *S. officinarum* (*So*)) are indicated by the proportional size of the pie diagrams, relative to expectations of *n *+ *n* inheritance. Cultivar names for each cross of the pedigree are provided in single quotes. ‘*’ indicates ‘2*n*’ chromosome transmission in the first two generations, and ‘+’ denotes an F_1_ hybrid. Although the exact pedigree of cultivars ‘R331’ and ‘Co213’ is unknown, they are estimated to be a BC2F2 and BC2:BC1 F_1_, respectively. IBD, identical by descent. **c**, Chromosome preparation of R570 after in situ hybridization, with *S. spontaneum*-specific probes shown in red. **d**, Karyotype diagram of R570 mirroring the colours in **b**.

The complexity of hybrid sugarcane genomes and pedigrees is exemplified by the development of the ‘R570’ cultivar, which was generated by breeders on Reunion island in 1980 (ref. ^11^) (Fig. 1a,b). Similar to other modern cultivars, R570 has a genome size (2 C) of approximately 10 billion bases (‘gigabases’ (Gb)), a ploidy of approximately 12*x* and 2*n* ≈ 114 chromosomes, several of which have recombined between progenitor species’ genomes^12,13^ (Fig. 1c,d); however, aneuploidy is common and the number of copies of each chromosome varies within and among cultivars. R570 was chosen as a model by the sugarcane community to study modern genome architecture and durable resistance to brown rust (*Puccinia melanocephala*), once a major disease in the tropics and subtropics^14,15^. Despite development of numerous R570 genetic resources (for example, cytogenetics, genetic maps, BAC clone libraries, ‘monoploid’ assembly^16^) and other attempts to assemble other cultivars^17,18^, modern sugarcane cultivars still lack a high-quality polyploid reference genome.

A genome such as R570 poses many technical assembly and genome representation challenges, as R570 has all the complexities of both outbred and inbred genomes. Given variable pairing affinities among R570 chromosomes, it could potentially be biologically appropriate to follow the standard outbred genome representation where an assembly is built for each meiotic homologue. However, given its backcrossed pedigree, 2*n* + *n* chromosome transmission and double maternal/paternal grandparent ‘POJ2878’ (Fig. 1b), we expect a majority of the genome to be inbred, with on average 12.5% of sequences exactly duplicated. Normally, identical sequences in inbred genomes are represented as a single collapsed haplotype (for example, the CHM13 human cell line^19^) or computationally duplicated in each haplotype (for example, tetraploid potato genome^20^). In the case of R570, it is impossible to confidently place exactly duplicated sequences due to variable copy number and complex patterns of recombination between progenitor subgenomes. Therefore, we opted for a standard partial-inbred genome assembly for R570, where the ‘primary’ assembly is a complete representation of unique haplotypes in R570 whereas the ‘alternate’ represents nearly identical, additional haplotypes. While ‘alternate’ here does not have the same meaning as compared to organisms with strict disomic pairing, we structured the R570 genome in a similar manner to improve utility for the community.

**Fig. 2:**
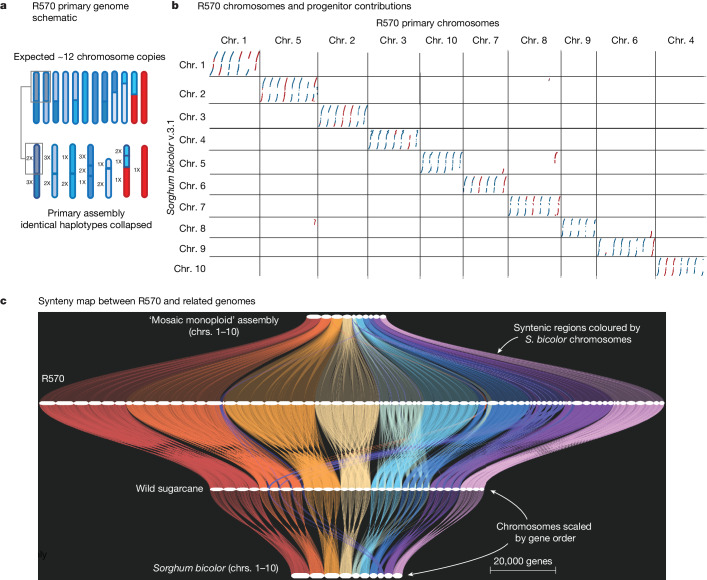
The genome assembly of sugarcane cultivar R570. **a**, Schematic representation of the primary genome assembly. Although R570 has approximately 12 chromosome copies per homolog, backcrossing and 2*n* + *n* chromosome transmission have led to near-identical haplotypes that are collapsed (represented as colour shades) in the genome assembly. **b**, One-to-one ortholog genes among chromosomes 1–10 of *Sorghum bicolor* (v.3.1.1) and primary chromosomes of R570. Each region is coloured based on progenitor contribution within R570. **c**, GENESPACE-generated synteny map among (bottom to top) *Sorghum bicolor* (v.3.1), *S. spontaneum* (genotype AP85-441), R570 primary and R570 monoploid genome assemblies. Horizontal segments indicate chromosomes; colours (red–purple) indicate the orthologous *Sorghum bicolor* chromosomes (1–10) and ‘braids’ represent syntenic blocks between each pair of genomes. *x-*axis positions are scaled by gene-rank order. Source Data

**Extended Data Fig. 1:**
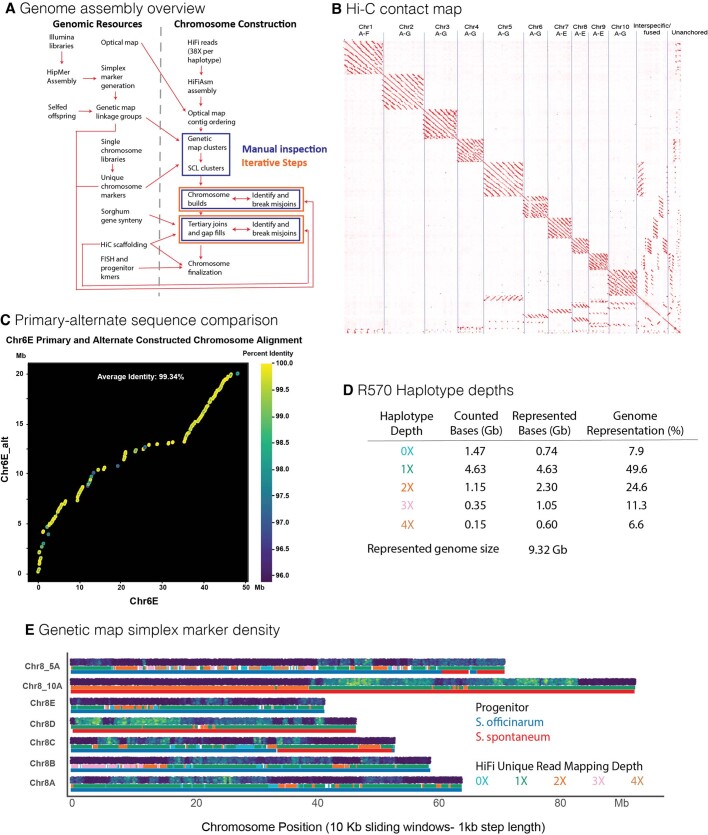
R570 genome assembly overview and analysis. A) Simplified genome assembly pipeline for sugarcane R570. The pipeline combines multiple sequencing technologies (HiFi contigs, optical map, genetic map, single chromosome libraries, Hi-C, Sorghum synteny), leveraging the strengths of each for phasing homeologous chromosomes, while using manual inspection and iterative steps to overcome each technology’s weaknesses. See ‘Supplemental Data’ for a full explanation and rationale for each step. B) Hi-C read heatmap for R570. Chromosomes are clustered based on homology, with Hi-C reads (~ 56X coverage) aligned to the finalized version of the assembly. C) Alignment of primary and alternate chromosomes. During genome construction and finalization, near perfect duplicate contigs were discovered when constructing chromosomes, which were binned into an alternate assembly to improve the utility of the genome (see Supplemental Data). When possible these contigs were ordered and oriented into chromosomes based on their closest, unique alignment in the primary assembly. For example, Chromosome 6E and its anchored alternate were aligned using nucmer (v4.0; -l 100 --maxmatch -b 400). Dots represents a 1-1 alignment between the two sequences that is greater than 15 kilobases in length. Each point is coloured based on alignment percent identity. D) Haplotype collapse summary for R570. To quantify regions of haplotype collapse within the R570 genome, PacBio HiFi reads were aligned to the assembly, using read depth to determine haplotype copy number. 0X unique coverage regions represent genomic blocks where reads could not uniquely map between the primary and alternate assembly. Counted bases represent genome sequences where depth could be reliably calculated (0-4X). Represented bases account for collapsed bases in the assembly (example 1 Mb of 3X coverage = 3 Mb represented sequence). E) Simplex marker densities in the sugarcane R570 genome. Simplex markers (80 bp) from the R570 genetic map (obtained by testing 3:1 segregation pattern in 96 genotyped S1 progeny) were searched in the R570 genome assembly, retaining only exact, single copy match locations. Position densities were then visualized by calculating the percent number of matched bases per 10 kb sliding window (1 kb step length). Lines underneath each chromosome correspond to progenitor and haplotype collapse block assignments.

In a typical genome, a highly contiguous assembly could be organized (‘scaffolded’) into chromosomes solely by Hi-C or optical mapping; however, both of these technologies require short unique sequence anchors, which are rare in the R570 genome. Therefore scaffolding required a custom pipeline that leveraged multiple lines of evidence, including PacBio HiFi circular consensus sequencing, Bionano Direct Label and Stain optical mapping, genetic linkage mapping, synteny, single-chromosome sorted sequencing and Hi-C. We combined these diverse resources through a custom pipeline (Extended Data Fig. 1a,Supplementary Data, Supplementary Figs. 1–11 and Supplementary Table 1) to construct a 5.04 Gb (12.6 Mb contig N50; average 12 contigs per chromosome) primary assembly (Fig. 2a,b, Extended Data Fig. 1b and Supplementary Fig. 12) that encompasses roughly half of the 10 Gb of sequence and 114 chromosomes (Methods) expected from R570 flow cytometry estimation^13^. The 3.7 Gb of additional sequence represented in the ‘alternate’ assembly are nearly identical to, but not necessarily meiotic pairs of, the corresponding primary chromosomes. For example: Chr6E_alt (20.4 Mb) is 99.34% similar to Chr6E (50.1 Mb; Extended Data Fig. 1c), and HiFi reads cannot be mapped uniquely to 39.7% of the alternate assembly (Supplementary Table 2). In addition to this highly similar sequence, R570 has an expected approximately 12.5% inbreeding coefficient due to a shared grandparent (POJ2878; Fig. 1b). Thus, we expect approximately 1.25 Gb of genome to be absent in the alternate assembly and collapsed to a single representation in the primary. Our 8.72 Gb combined primary and alternate assembly very closely aligns with this expectation.

The high-quality (0.1% gaps; long terminal repeat (LTR) assembly index (LAI)^21^: 22.82) primary assembly captures a full representation of the diversity present in R570 and will serve as the basis for genome-enabled biotechnology in sugarcane. As is the case with typical outbred diploid genomes, duplicate copies between haplotypes can complicate or bias analyses—usually one haplotype is used as the reference for mapping. Thus, here we focus on the primary assembly for efforts central to candidate gene discovery, such as gene expression and variant detection. To support these efforts, we used gene homology and RNA sequencing (RNA-seq) transcript evidence to describe the full suite of protein coding sequences and annotate genes in the primary R570 assembly. The primary annotation is highly complete (BUSCO = 99.8% total, 99.3% duplicate completeness)^22^ with 194,593 coding sequences (and 105,138 alternative spliced transcripts). In contrast to previous monoploid assemblies, which contained a single representation of each ancestral chromosome, synteny-aware gene families (built with GENESPACE^23^) were present in six (*n* = 40,752) copies in the primary genome (6.78 mean syntenic block coverage with *Sorghum bicolor* (*S. bicolor*); Fig. 2c, Table 1 and Supplementary Table 3), which reflects half of the expected 12*x* ploidy and matches the expected copy number in the primary assembly. This within-genome variation is now available to breeders, but was obscured with current monoploid (single-copy) methods. Combined, the primary and alternate assemblies provide by far the most complete genomic sequences available for cultivated sugarcane.

**Table 1:** R570 genome assembly and annotation statistics

	Overall	*S. officinarum*	*S. spontaneum*
Primary genome size (contig N50)	5.04 Gb (12.6 Mb)	3.66 Gb	1.37 Gb
Alternate genome size (contig N50)	3.73 Gb (2.1 Mb)	3.01 Gb	0.32 Gb
Genome size accounting for collapsed haplotypes	9.32 Gb	-	-
Collapsed haplotypes in assembly^a^	2.31 Gb	2.18 Gb	0.116 Gb
No. of genes (no. of syntenic orthogroups) in primary annotation^b^	194,593 (47,986)	132,618	61,197
Mean ploidy of primary assembly (coverage of syntenic blocks)^b^	6.78*x*	4.60*x*	2.16*x*
Mean pairwise peptide identity among alleles^c^	-	86%	83%
Genes impacted by structural variants^d^	5,362	5,090	260

^a^HiFi unique mapping ≥ ×2 expected depth. ^b^GENESPACE default parameters. ^c^Calculated among peptides from the primary annotation within syntenic orthogroups. ^d^Calculated from pairwise alignments, relative to ChrA among homologous chromosomes.

## The architecture of the R570 genome

Knowledge of the global genome architecture of modern sugarcane cultivars is currently derived mainly from molecular cytogenetics^12,13,24,25^, genetic mapping^8,16,26^ and haplotype sequence comparisons^27–30^. Our chromosome-scale R570 assembly provides the first fine-grain description of the genome architecture of modern sugarcane cultivars, a foundation to describe the patterns of genomic evolution and diversity within a neo-polyploid hybrid, a crucial resource for burgeoning sugarcane molecular breeding efforts. Perhaps the most critical element of interspecific sugarcane breeding is the maintenance and enrichment of *S. spontaneum* progenitor sequence, conferring disease resistance and environmental adaptation^25^. The progenitor species of R570 are highly diverged (approximately 1.6 million years; Supplementary Table 4 and Supplementary Fig. 13), which enabled extraction of 27 bp species specific repeats used to assign progenitor blocks in the genome (Supplementary Data). Consistent with previous cytogenetic estimates^12,13^, we found that 3.66 Gb (73%) and 1.37 Gb (27%) of the R570 primary genome assembly (5.04 Gb) is derived from *S. officinarum* and *S. spontaneum*, respectively (Supplementary Tables 5 and 6). Separate evolutionary trajectories have also produced distinct ploidy levels and basic chromosome numbers between progenitors (*S. officinarum,* 2*n* = 8*x*, basic chromosome number *x* = 10; *S. spontaneum,* 2*n* = 4 − 16*x* = typical basic chromosome number *x* = 8). The basic chromosome set (*x* = 10) of *S. officinarum* is directly syntenic to the ten chromosomes of *S. bicolor*, its most well-studied annotated diploid relative. In contrast, the basic chromosome set (*x* = 8, but can vary) of *S. spontaneum* is a result of six chromosomes being rearranged into four^13,16,31,32^, each of which are observed in the R570 primary assembly (Chr5_9A, Chr 6_9A, Chr 7_10A and Chr 8_10A; Fig. 2b).

Despite rearrangements in *S. spontaneum*, most of the progenitor chromosomes within R570 are syntenic and share sequence homology, facilitating interspecific recombination. Indeed, cytogenetic experiments among multiple sugarcane hybrid cultivars indicate that homologous pairing and recombination between chromosomes from different progenitors is likely common^12,25^. In the R570 primary assembly, we observed 13 interspecific recombinant chromosomes among seven of ten basic chromosomes (Fig. 2b). The assembly also confirmed a cytogenetic predicted chromosome resulting from a translocation between *S. spontaneum* chromosome 5 and *S. officinarum* chromosome 8 (Fig. 2b) which is so far found only in R570 and no other modern cultivar^13^. Homoeologous introgressions, which can be enriched in breeding targets, have been observed in other systems, both in traditional breeding (for example, oat^33^) and synthetic polyploids (for example, Brassica^34^ and wheat^35^). R570 recombinant chromosomes contain diversity within progenitor genomes that is not easily purged through inbreeding, likely providing additive genetic variance accessible to breeders in advanced-generation intercrosses.

Breeding practices such as backcrossing, ‘2*n*’ chromosome transmission and small breeding population sizes, have resulted in high DNA sequence redundancy and exact duplicates, particularly those derived from *S. officinarum*. For example, the cultivar ‘POJ2878’ has been used in many breeding programs worldwide and is both a maternal and paternal grandparent of R570 (Fig. 1b). To catalogue the genomic structure of copy number variation and molecular sequence variation within R570, we used highly accurate PacBio HiFi reads (median length 17 kb), to find roughly half the genome (50.4%) is identical-by-descent where haplotypes are collapsed among multiple copies (2–4*x*) (Supplementary Table 7, Supplementary Fig. 14 and Extended Data Fig. 1d). The remainder of the genome (49.6%) contains enough sequence variation (heterozygosity) to enable single, unique alignments of PacBio reads that distinguish separate haplotypes. Each of basic chromosomes of R570 are covered by one to four *S. spontaneum* haplotypes (Fig. 2b) most of which (86%) is heterozygous, single-copy sequence. In contrast, only 48% of the *S. officinarum* portion is heterozygous, while the majority is collapsed among multiple haplotypes. Indeed, 87% of the duplicated sequence among the primary and alternate assemblies (39.7%; previously discussed; Supplementary Table 2) is derived from *S. officinarum*. Since breeding for increased sugar content and other traits rely on additive contributions of gene dosage, these perfectly duplicated regions represent potential targets for copy-number aware genotyping and molecular breeding efforts. However, exploring the genomic contribution of the domesticated progenitor is difficult as genotyping inbred haplotypes require restrictively large numbers of progeny to screen (for example, triplex marker segregation in S1 = 143:1 (ref. ^36^)). The most common genetic marker used for sugarcane breeding (simplex, segregation in S1 = 3:1 (ref. ^37^); Supplementary Data) is significantly biased toward the *S. spontaneum* regions of the genome (45% of markers; Fisher exact test: ×3.25 enrichment, *P* < 0.0001), and is found almost exclusively in heterozygous haplotypes (98%) (Extended Data Fig. 1e). While this bias towards heterozygous regions renders the majority of the genome invisible to traditional genetic mapping, the R570 assembly will allow easier exploration of quantitative trait loci (QTLs) through cataloguing of haplotype structure and progenitor contribution within the genome.

## Exploration of targets for breeding

Many crucial traits for sugarcane improvement are polymorphic in the progenitor species and dosage dependent in hybrid breeding programs. For example, brown rust resistance (see below) appears to be derived from a single-copy locus within the genome, while high sugar content requires additive contributions of gene copies from *S. officinarum*. To accelerate similar breeding efforts and develop marker assisted selection strategies, we documented copy number and protein sequence variation between and within R570 progenitor subgenomes within the primary assembly and annotation (Table 1, Fig. 2c and Supplementary Table 3). Using progenitor block classification, we were able to assign 68% of gene models (*n* = 132,618) to *S. officinarum* and 31% to *S. spontaneum* (*n* = 61,197). Inspection of homeologs among progenitors found 87% of gene copies derived from *S. officinarum* and 95% derived from *S. spontaneum* contained non-synonymous variation (Supplementary Table 8), but it is important to note that many of these genes are located in regions where haplotypes are collapsed (*n* = 58,038; 87% *S. officinarum* assigned; Supplementary Table 9), and thus some gene models are likely under-represented. Peptide polymorphism largely mirrored the % identical homeolog analyses, where *S. officinarum* homeologs had an average pairwise identity (PID) of 86% while *S. spontaneum* homeologs had significantly more variation (mean PID = 83%; Mann–Whitney *U* = 3.5 × 10^8^, *P* < 0.0001). The investigation of genes impacted by structural variants, which may prevent recombination and subsequent generation of desirable allelic combinations is also significantly biased towards *S. officinarum* portions of the genome (*n* = 5,090; 94% of impacted genes; Fisher’s exact test, odds ratio: 9.03, *P* < 0.0001; Supplementary Table 10). A survey of unique material (genes with no orthology in the other progenitor; *n* = 32,544) found ×1.2 more genes derived from *S. officinarum* than expected (Fisher’s exact test, odds ratio: 1.24, *P* < 0.0001); although investigation of the largest novel gene family contributed from the *S. spontaneum* found a nine gene tandem duplication of leucine rich repeat genes on Chr7_10A. Furthermore, annotation of resistance gene analogues (RGAs)^38^ throughout the genome (Supplementary Table 11) showed significant enrichment for *S. spontaneum* derived motifs (Fisher’s exact test, odd’s ratio 2.14, *P* < 0.0001), particularly on homologous regions of chromosomes 3, 6 and 7 (×4.81, ×3.35 and ×4.11 enrichment, respectively, *P* < 0.0001; Supplementary Table 12).

Hybrid and backcrossing breeding programs often introduce large swaths of linked maladaptive alleles that reduce crop yield in early generations. In modern sugarcane cultivars, interspecific hybridization not only introduced disease resistance alleles from *S. spontaneum*, but also alleles that reduced the high-sucrose (‘brix’) content in the domesticated *S. officinarum*. Previous studies suggested that discrete loci disproportionately explained sugar content variation^39–41^, but some of these experiments were performed in different genetic backgrounds, with only the monoploid assembly or *S. bicolor* available for candidate gene discovery, offering a collapsed view of allelic variation that exists in the R570 genome. Using comparative genomics between *S. bicolor* BTx623 (short stature, early maturing, cereal genotype) and rio (‘sweet sorghum’; tall, late maturing, high soluble sugar content), we explored sugar transport genes underlying the rio ‘sweet’ phenotype of high concentrations of soluble sugars within its stem^42^, a phenotype also of interest by sugarcane breeders. Of the candidates described in ref. ^42^, 43 *S. bicolor* BTx623 genes were contained as single placement anchors within R570 syntenic orthogroups, with 505 syntenic orthologs among other genomes (*Sorghum* ‘rio’: R570 monoploid: *S. spontaneum* (genotype AP85-441): R570; syntenic orthologs per genome = 39:37:130:299; mean gene copies per homologue per genome = 1:1:3:7).

**Extended Data Fig. 2:**
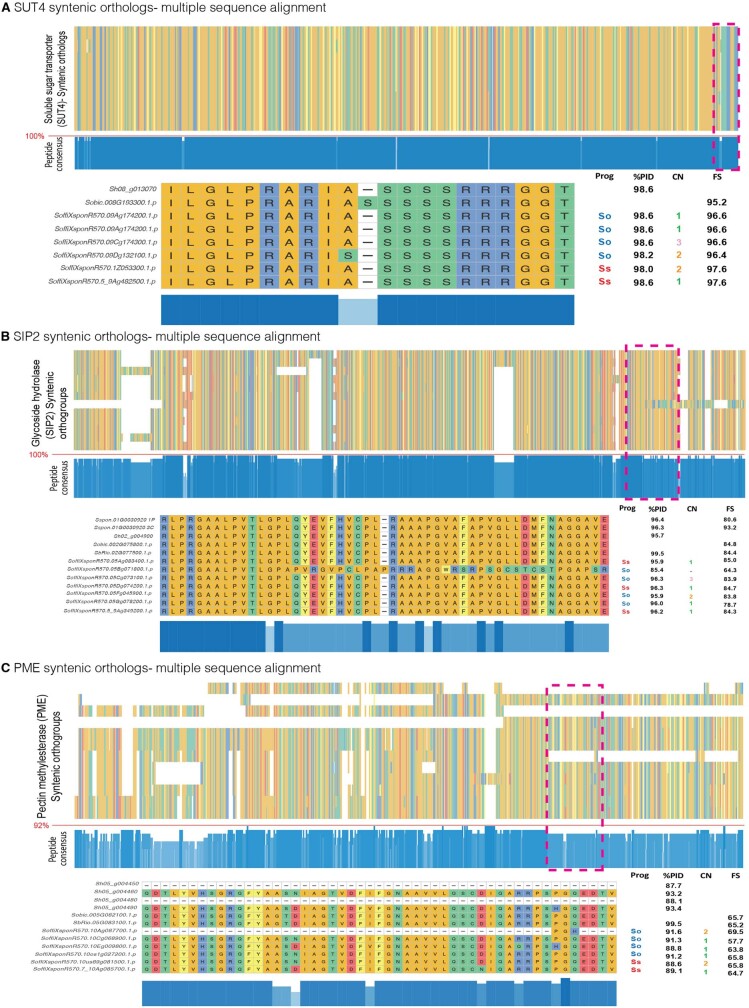
Sugar accumulation candidate gene alignments. A) Alignment of syntenic orthologs for SUT4 (Sobic.008G193300). B) Alignment of syntenic orthologs for SIP2 (Sobic.002G075800). C) Alignment of syntenic orthologs for PME (Sobic.005G082100). Top section for each panel shows the full length of the alignment, while the bottom section displays a zoomed in region (outlined in a pink dotted outline) to show specific differences among species and alleles. Prog- progenitor assignment of R570 alleles. Each ortholog position was intersected with progenitor assignments to assign origin to each peptide. %PID- Percent pairwise identity (number of matches/alignment length * 100) for each syntenic ortholog relative to the *S. bicolor* (BTx623). Copy number (CN)- PacBio HiFi read depths (representing collapsed identical haplotypes) ranges were intersected with gene position to provide an indication of additional collapsed alleles (eg. copy number = 2; one represented allele + one identical collapsed allele). Fold score (FS)- syntenic orthologs (except for monoploid annotated peptides) were folded using ESMfold and scored for quality. Score provided is the percent of high-quality amino acids present in the peptide sequence.

Percent PID among the *S. bicolor* homologue and syntenic orthologs found sugar transport genes are highly conserved (*Sorghum* ‘rio’: R570 monoploid: *S. spontaneum* (genotype AP85-441): R570; median PIDs per genome = 100%:91%:94%:94%) (for example, SUT4-Sobic.008G193300, Extended Data Fig. 2a), although some R570 alleles contain frameshift mutations that are likely to impair function (for example, SoffiXsponR570.05Bg071800-L744A-Sobic.002G075800-Glycoside hydrolase ortholog, *S. officinarum* allele, Extended Data Fig. 2b) or possess highly variable alleles with regions where individual homeologs can be distinguished (for example, Sobic.005G082100-cell wall pectinesterase; Extended Data Fig. 2c). Annotation of the R570, paired with information of gene dosage, allelic variation and progenitor contribution will enable the sugarcane community to better comprehend germplasm resources at their disposal, for both R570 and other hybrid cultivars.

**Fig. 3:**
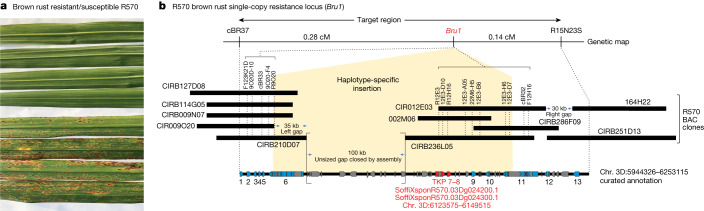
*Bru1* candidate gene locus. **a**, Brown rust disease resistance in R570. Top panel shows selfed R570 offspring with the *Bru1* locus, while the bottom panel shows offspring lacking *Bru1*. **b**, Gap-filled haplotype assembly identifies a TKP as candidate causal genes for *Bru1* durable brown rust resistance. Blue pentagons represent curated gene models and grey pentagons are large transposable elements. *Bru1* TKP7 and TKP8 candidate genes are indicated in red with their location on Chr. 3D.

Apart from high sugar production, a defining characteristic of modern sugarcane cultivars is biotic disease resistance. One of the most important diseases that affects all sugarcane growing regions around the world is brown rust, caused by the fungus, *Puccinia melanocephala*. Once a major pathogen of sugarcane that caused yield losses of up to 50%, breeders have successfully mitigated *P. melancocephala*-derived losses by selecting for disease resistance. A major locus (*Bru1*) that confers durable resistance to this disease (Fig. 3a) was identified in cultivar R570 (refs. ^43,44^). To uncover the causative allele underlying *Bru1*, previous studies used an extensive map-based cloning approach that screened approximately 2,400 self-pollinated R570 progeny, constraining *Bru1* to a set of BAC sequences that spanned approximately 209 kb (refs. ^27,44^) (Methods). Although the region contained 13 gene models (Fig. 3b and Supplementary Table 13), it also contained an unsized gap and large haplotype insertion, both of which prevent further fine-scale mapping and exhaustive candidate gene discovery^27,44^. Nonetheless, the fixed insertion haplotype enabled the design of *Bru1* diagnostic PCR markers. These have been effectively used in modern cultivar breeding programs worldwide, demonstrating that the single-dose *Bru1* locus has been the major source of effective (or ‘durable’) brown rust resistance for decades across multiple environments^14^.

In contrast to previous resources, our R570 genome assembly spans the entirety of the *Bru1* target region (chromosome 3D: 5944326–6253115 bp). Crucially, this includes a complete approximately 100 kb stretch of contiguous sequence across the previously unsized gap region^44^. Filling this previously unsized gap and demonstrating that it did not include additional candidate genes was an essential step before investing in the analysis of all candidate genes in the region. Manual curation of the gap-filled region confirmed the 13 gene models, whose functions were assessed, searching for genes involved in disease resistance mechanisms, with two genes standing out as top candidates (Methods). Curated genes 7 and 8 (gene IDs, SoffiXsponR570.03Dg024200 and SoffiXsponR570.03Dg024300) share homology (both classified as RLK-PELLE-DSLV kinases^45^), are located within the bounds of the haplotype-specific insertion (Fig. 3b), and are each single copy in the R570 genome. While gene 7 (SoffiXsponR570.03Dg024200) contains all 12 functional kinase subdomains, gene 8 (SoffiXsponR570.03Dg024300) contains only domains I through VII and is likely a pseudokinase. These two genes represent a tandem kinase-pseudokinase (TKP), similar to barley stem rust (RPG1 (ref. ^46^)) and yellow rust resistance Yr15 (ref. ^47^). The current model of molecular action for TKP resistance suggests the pseudokinase acts as a decoy for fungal pathogen effectors^48^, while the functional kinase generates a signal cascade, innervating the plant effector-triggered immune response. Due to their variation and novelty, TKPs (and other variants (for example, tandem kinase-kinases and so on)) are difficult to find using only sequence homology. Their structure has been predicted across the plant domain of life, but only five examples have been functionally validated in monocots, all of which conferred resistance to fungal pathogens^49^. Combined, these results support this tandem kinase-pseudokinase (TKP7 and TKP8) as the causal gene for *Bru1* brown rust resistance and will permit future biotechnological improvement of sugarcane for brown rust.

## Conclusions

The polyploid genome assembly and annotation of sugarcane cultivar R570 is an essential stepping stone in the emerging genomic revolution for sugarcane. This work reveals the genomic effects of breeding practices that transformed sugarcane into sugar/biomass production factories, a remarkable feat by breeders considering the complexity of the genome and the revelation that much of the ‘sweet’ domesticated alleles contributed from *S. officinarum* are identical and thus are largely inaccessible to QTL mapping efforts. Further, the persistence of the *S. spontaneum* progenitor genomic contribution, despite multiple rounds of backcrossing to *S. officinarum* and 2*n* + *n* chromosome transmission, is highlighted by the enrichment of both RGA motifs and unique gene family contributions from the wild progenitor species. The ability to separate, resolve and investigate individual haplotypes and chromosomes within R570 enables a much greater understanding of the fine-grain architecture of this very complex genome and will lead to substantial improvements in the genetic understanding of agronomic traits through exploration of allelic variation, copy number and gene presence/absence variation^2^.

One of the most important, yet complex, questions underlying agronomic trait discovery in sugarcane is epistatic interaction among alleles. Desirable traits such as sucrose transport and accumulation are complex enough in diploid plants, let alone in highly polyploid sugarcane with approximately 12*x* copies of each chromosome. Annotation and pan-genome synteny networks in R570, paired with new differential expression analyses enabled by this work, will help reveal the complicated regulation of transcription factors and multiple, identical target sequences within sugarcane. Furthermore, demonstrating that while half the genome is identical/collapsed among haplotypes, the remaining sequence is heterozygous and is over-represented by *S. spontaneum* will help improve the construction and design of genetic markers that do not rely solely on segregation for QTL mapping. While interspecific hybrid sugarcane represents one of the most complex plant genomes ever sequenced, it is likely by no-means the most complex genome that kingdom Plantae can offer. The strategies outlined here that combine multiple sequencing technologies and techniques are broadly applicable and can be applied to complex plant genomes sequenced in the future. Description of the *Bru1* disease resistance locus and discovery of strong candidate genes corresponding to a tandem kinase-pseudokinase will allow targeted validation experiments. Its putative molecular function supports that tandem kinase resistance mechanisms are durable and capable of protecting globally distributed crops across many environments. This work represents the culmination of a decades-long global collaboration by sugarcane breeders and researchers to develop genomic resources for R570 to better understand one of the most valuable crops in the world, the modern sugarcane hybrid cultivar.

## Methods

### Genome sequencing

#### Illumina libraries

Illumina libraries for this manuscript were sequenced on a combination of Illumina X10, HiSeq and NovaSeq platforms. HipMer assembly and selfed progeny (Extended Data Fig. 1a): sequencing libraries were constructed using an Illumina TruSeq DNA PCR-free library kit using standard protocols. Libraries were sequenced on an Illumina X10 instrument using paired ends and a read length of 150 base pairs.

##### Single flow-sorted chromosome libraries

Sequencing libraries were constructed using an Illumina TruSeq DNA Nano library kit using standard protocols. Libraries were sequenced on either the Illumina HiSeq2500 or NovaSeq 6000 instrument using paired ends and a read length of 150 base pairs.

##### Remaining Illumina libraries

Illumina Tight Insert Fragment, 400 bp–2 ug of DNA was sheared to 400 bp using the Covaris LE220 and size selected using the Pippin (Sage Science). The fragments were treated with end-repair, A-tailing and ligation of Illumina compatible adaptors (IDT) using the KAPA-Illumina library creation kit (KAPA Biosystems). The prepared libraries were quantified using KAPA Biosystems’ next-generation sequencing library qPCR kit (Roche) and run on a Roche LightCycler 480 real-time PCR instrument. The quantified libraries were then prepared for sequencing on the Illumina HiSeq sequencing platform using a TruSeq Rapid paired-end cluster kit, v.2, with the HiSeq 2500 sequencer instrument to generate a clustered flowcell for sequencing. Sequencing of the flowcell was performed on the Illumina HiSeq 2500 sequencer using HiSeq Rapid SBS sequencing kits, v.2, following a 2 × 250 indexed run recipe.

#### PacBio libraries

Continuous long-read PacBio sequencing primer was then annealed to the SMRTbell template library and sequencing polymerase was bound to them using a Sequel Binding kit v.2.1. The prepared SMRTbell template libraries were then sequenced on a Pacific Biosystem Sequel sequencer using v.3 sequencing primer, 1 M v.2 single-molecule real-time cells and v.2.1 sequencing chemistry with 1 × 600 sequencing video run times. PacBio HiFi sequencing was performed using circular consensus sequencing (CCS) mode on a PacBio Sequel II instrument. High molecular weight DNA was either needle-sheared or sheared using a Diagenode Megaruptor 3 instrument. Libraries were constructed using SMRTbell Template Prep Kit v.2.0 and tightly sized on a SAGE ELF instrument (1–18 kb). Sequencing was performed using a 30 h video time with 2 h pre-extension and the resulting raw data was processed using the CCS4 algorithm.

#### RNA-seq libraries

Illumina RNA-Seq with poly(A) selection plate-based RNA sample preparation was performed on the PerkinElmer Sciclone NGS robotic liquid handling system using Illumina’s TruSeq Stranded mRNA HT sample prep kit using poly(A) selection of mRNA following the protocol outlined by Illumina in their user guide: https://support.illumina.com/sequencing/sequencing_kits/truseq-stranded-mrna.html, and with the following conditions: total RNA starting material was 1 ug per sample and eight cycles of PCR were used for library amplification. The prepared libraries were quantified using KAPA Biosystems’ next-generation sequencing library qPCR kit and run on a Roche LightCycler 480 real-time PCR instrument. Sequencing of the flowcell was performed on the Illumina NovaSeq sequencer using NovaSeq XP v.1 reagent kits and an S4 flowcell, following a 2 × 150 bp indexed run recipe.

#### Chromosome in situ hybridization

Chromosome mitotic metaphase preparations and fluorescence in situ hybridization were performed as described in ref. ^13^. The *S. spontaneum* retro-transposon specific oligo probe was designed by Arbor Biosciences using their proprietary software based on the retro-transposon sequences as described in ref. ^50^. Probes were either labelled with fluorochromes ATTO 488 or ATTO 550.

#### Single flow-sorted chromosome preparation

Stems of adult plants were cut into single-bud segments, cleaned and soaked in 0.5% carbendazim solution for 24 h, placed in a plastic tray, covered with wet perlite and incubated at 32 °C in the dark, until the roots were approximately 1.5 cm long. For cell-cycle synchronization and accumulation of metaphases, the segments were washed in ddH_2_O, then transferred to a plastic tray filled with 150 ml 0.1 × Hoagland solution containing 3 mmol l^−1^ hydroxyurea and incubated at 25 or 32 °C for 18 h in the dark. After a 2 h recovery treatment, the roots were immersed in 2.5 µmo l^−1^ amiprophos-methyl solution and incubated for 3 h at 25 or 32 °C. Suspensions of intact chromosomes were prepared by mechanical homogenization of root tips fixed with 3% formaldehyde and 0.5% Triton X-100, and stained with 4′,6-Diamidino-2-phenylindole dihydrochloride (DAPI)^51^. The instrument used for flow sorting was a FACSAria II SORP flow cytometer (BD Biosciences) and Beckman Coulter MoFlo AstriosEQ cell sorter (Beckman Coulter). The software used was FACSDiva v.6.1.3 (BD Biosciences) and Summit v.6.2.2 (Beckman Coulter). For chromosome sorting, initial gating was set on dotplots DAPI-A versus FSC-A and the final sorting gate was set on DAPI-A versus DAPI-W dotplots to exclude chromosome doublets (Supplementary Fig. 15). The identity of flow-sorted fractions was determined by fluorescence microscopy of chromosomes sorted onto microscope slides^51^. The analysis revealed that chromosomes could be separated into a few size fractions and while the sorted populations were 100% pure chromosomes, it was not possible to sort individual sugarcane chromosomes. To overcome this problem and prepare samples of chromosome-specific DNA for sequencing, single copies of chromosomes were sorted and their DNA amplified^52^. This strategy for preparing sugarcane chromosomes for flow cytometry was first described in ref. ^51^ and is a modification of the protocol described in ref. ^53^.

#### Optical map construction

Ultra-high molecular weight (uHMW) DNA was isolated from agarose-embedded nuclei as previously described in ref. ^54^ with some modifications. Approximately 2 g of young, healthy R570 leaves were collected and fast-frozen in a 50 ml conical tube, ground in a mortar with liquid nitrogen and briefly incubated in Bionano homogenization buffer (HB+; Bionano Plant DNA isolation Kit; Bionano Genomics). Cell debris was filtered out by sequentially passing the homogenate through 100 µm and 40 µm cell strainers. Nuclei in suspension were pelleted by centrifugation at 2,000*g* at 8 °C for 20 min, resuspended in 3 ml homogenization buffer HB+ and subjected to discontinuous density gradient centrifugation as described in the Plant Tissue DNA Isolation Base Protocol (Revision D; Bionano Genomics). The nuclei-enriched interphase layer was recovered, pelleted and embedded in low-melting-point agarose using a 90-µl CHEFgel electrophoresis plug mould (Bio-Rad). The resulting plug was incubated twice, for a total of 12 h at 50 °C, in Bionano Lysis buffer supplemented with 1.6 mg ml^−1^ Puregene Proteinase K, washed four times in Bionano Wash Buffer and five times in TE buffer. The uHMW nDNA was recovered by melting and digesting the plug with agarase at 43 °C, followed by drop dialysis. In total, approximately 9 µg uHMW DNA was recovered at a concentration of 136 ng µl^−1^ and used for subsequent genome mapping processes.

Genome mapping was performed using the Bionano Genomics Direct Label and Stain chemistry in a Bionano Saphyr instrument, using the method described in ref. ^55^, with a few modifications. Approximately 800 ng of uHMW DNA was used per reaction and a total of eight flow cells were loaded to collect molecules with a total combined length of 3,499,160 Mbp. A subset of 1,650,737 molecules with a minimum length of 450 kb, and N50 of 547 kb were selected for assembly. The final total combined length of the filtered subset was 1,097,878,758 bp, with estimated effective coverage of assembly of ×101.2.

Genome assembly was performed using the Bionano Genomics Access software platform (Bionano Tools v.1.3.8041.8044; Bionano Solve v.3.3_10252018), running the pipeline v.7981 and RefAligner v.7989. Two separated assemblies were performed using the optArguments_nonhaplotype_noES_BG_DLE1_saphyr.xml parameters. The initial assembly was performed without complex multi-path region (CMPR) cuts and produced 570 maps with a N50 length of 36.444 Mbp and total map length of 7,654.039 Mbp. One additional assembly was performed using the CMPR cut option, which introduces map cuts at potential duplications to reduce potential homeolog and phase switching. CMPR-cut-enabled assembly generated 1,512 maps with N50 length of 9.546 Mbp and total map length of 9,282.351 Mbp.

PacBio HiFi Bionano hybrid scaffolds were generated using the Bionano Genomics Access software (Tools v.1.3) and the DLE-1 configuration file hybridScaffold_DLE1_config.xml using auto-conflict resolution. In total, the genome was captured in 122 hybrid scaffolds (Scaffold N50 = 78.823 and maximum scaffold size of 131.769 Mbp. The total scaffold length was 5,074 Mbp, with 4.9 Mbp of sequence remaining un-scaffolded.

#### Genome assembly overview

Complete representation of all sequences in the 10 Gb genome of R570 was impossible without artificially duplicating collapsed sequences, of which there are many. To scaffold the contigs into chromosomes, we applied five complementary techniques (Supplementary Data). First, we used the Bionano optical map to initially order contigs into long-range scaffolds. Second, scaffolds were clustered into homeologous groups based on 237 linkage groups constructed from approximately 1.8 million simplex markers that were assayed from 96 self-pollinated progeny. Third, additional clustering was performed using genetic markers derived from single flow-sorted chromosome libraries sequenced from R570 (refs. ^52,53^). After making initial joins, both simplex and single-chromosome genetic markers were re-aligned putative chromosomes to investigate misjoins, which were broken and corrected. Fourth, we resolved overlapping scaffolds by checking for redundant collinear sets of *Sorghum bicolor* gene models mapped against the contigs using pblat^56^ with default parameters. Finally, we manually evaluated chromatin linkages from 558 Gb (approximately ×56) Hi-C data to manually verify joins made between scaffolds during chromosome construction (Extended Data Fig. 1a). The highly contiguous primary assembly (5.04 Gb, 12.6 Mb contig N50; 67 chromosomes) also includes optical scaffolds (‘os’; *n* = 20) and unanchored scaffolds (*n* = 56). The primary assembly contains 0.1% gaps with an LTR assembly index^21^ (LAI; measure of intact LTR elements) of 22.82, indicating the assembly is high quality and complete. Where possible, the alternate assembly (3.73 Gb, 2.1 Mb contig N50; comprised of nearly identical haplotypes in the primary assembly; discussed in Supplementary Data), was physically anchored to the most similar chromosome in the primary assembly based on best unique alignments using minimap2 (v.2.20-r1061)^57^. Contigs and scaffolds that did not have a single best unique alignment were left unanchored. It should be noted that this sequence similarity-based grouping does not suggest that contigs on alternative scaffolds with the same name (for example, Chr6E and Chr6E_alt) necessarily come from the same biological haplotype. Thus, we provide the alternate scaffolds to represent the complete population of sequences in R570, and not as a source for global comparisons against the primary or other reference genomes.

#### Collapsed haplotypes

To determine which regions of the genome were perfectly identical and collapsed into a single haplotype (in contrast to the alternate assembly that contains nearly identical haplotypes, which could be distinguished by the assembler but most often not by unique HiFi read placements), PacBio HiFi reads were re-aligned back to the assembly using minimap2 (ref. ^57^) (parameters: -M 0 --secondary=no --hard-mask-level -t 30 -x asm5). Read coverage (script: combinePAFsAndCount.R) was calculated using script: relative to the median depth (37) per 10 kb window, ignoring repetitive regions where the median coverage was greater than five (greater than ×185 raw coverage). Depth classifications (×0–4) were calculated from the median coverage ranges (×0 (0–0.25), ×1 (0.25–1.4), ×2 (1.4–2.3), ×3 (2.3–3.5), ×4 (3.5–5.0)), based on histogram peaks. Depth classifications per 10 kb window were converted to their run-length equivalent using the script: convertCountsToRLEs.R. To ensure accurate representation of haplotypes, NucFreq^54^ was used to analyse regions where haplotypes were collapsed (×2–4 depth regions; approximately 1.2 Gb of primary genome sequence). In summary, HiFi reads were aligned to the combined primary and alternate assembly using pbmm2 (v.1.1.0; parameters: --log-level DEBUG --preset SUBREAD --min-length 5,000 --sort). Samtools^58^ was then used to merge individual bam files (from each HiFi sequencing run) and exclude unmapped reads and supplementary alignments. (samtools view -F 2308). The NucFreq output coverage bed (obed) file was converted to run-length equivalents (script: RLEruns.R), where alternate base calls were greater than 20% of the combined coverage. To ensure adequate coverage for analysis, regions with outlier depth ranges beyond the 10th and 90th percentiles were excluded. Additionally, repetitive regions of the genome (95% repetitive, masked with a 24mer and 10 kb regions where greater than 90% of bases were annotated as retrotransposons (from LAI analysis) were also excluded using BEDtools^59^ subtract. Of the approximately 1.2 Gb considered, approximately 4.8 Mb of sequence (0.4% of considered regions; 0.1% of bases within constructed primary chromosomes) appear to contain non-identically collapsed haplotypes, mainly driven by high depth collapsed regions (×2–3 depth regions = 0.3% of bases; ×4 depth regions = 1.5% of bases).

#### Genome annotation

Gene models were annotated using our PERTRAN pipeline (described in detail in ref. ^60^ using approximately 3.7 B pairs of 2 × 150 stranded paired-end Illumina RNA-seq and 31 M PacBio Iso-Seq CCSs reads. In short, PERTRAN conducts genome-guided transcriptome short read assembly via GSNAP (v.2013-09-30)^61^ and builds splice alignment graphs after alignment validation, realignment and correction. The resulting approximately 1.5 M putative full-length transcripts were corrected and collapsed by genome-guided correction pipeline, which aligns CCS reads to the genome with GMAP^61^ with intron correction for small indels in splice junctions if any and clusters alignments when all introns are the same or 95% overlap for single exon. Subsequently 1,763,610 transcript assemblies were constructed using PASA (v.2.0.2)^62^ from RNA-seq transcript assemblies above. Homology support was provided by alignments to 17 publicly available genomes and Swiss-Prot proteomes. Gene models were predicted by homology-based predictors, FGENESH+ (v.3.1.0)^63^, FGENESH_EST (similar to FGENESH+, but using expressed sequence tags (ESTs) to compute splice site and intron input instead of protein/translated open reading frames (ORFs) and EXONERATE (v.2.4.0)^64^, PASA assembly ORFs (in-house homology constrained ORF finder) and from AUGUSTUS (v.3.1.0)^65^ trained by the high confidence PASA assembly ORFs and with intron hints from short read alignments. We improved these preliminary annotations by comparing sequences and gene quality between R570 subgenomes by aligning high-quality gene models between subgenomes and forming gene models from intragenomic alignments. We compared scores between these intragenomic homology-based models and the PASA assemblies; higher-scoring homology supported models that were not contradicted by transcriptome evidence were retained to replace existing partial copy. The selected gene models were subject to Pfam analysis and gene models with greater than 30% Pfam TE domains were removed. We also removed (1) incomplete, (2) low-homology-supported without full transcriptome support and (3) short single exon (less than 300 BP CDS) without protein domain nor transcript support gene models. Repetitive sequences were defined using de novo by RepeatModeler (v.open1.0.11)^66^ and known repeat sequences in RepBase.

#### Comparative genomics

Syntenic orthologs among the R570 primary annotation, *S. bicolor* (v.3.1)^67^, *S. spontaneum* (genotype AP85-441)^32^, *Setaria viridis* (v.2.1)^68^ and the R570 monoploid path^16^ were inferred via GENESPACE (v.0.9.4)^23^ pipeline using default parameters (analysis script: genespaceCommands.R). In brief, GENESPACE compares protein similarity scores into syntenic blocks using MCScanX^69^ and uses Orthofinder (v.2.5.4)^70^ to search for orthologs/paralogs within synteny constrained blocks. Syntenic blocks were used to query pairwise peptide differences among progenitor alleles, determine divergence among progenitor orthologs using *S. bicolor* syntenic anchors and search for progenitor specific orthogroups (scripts, PID_calc.R; GENESPACE_orthogroupParsing.R; Jupyter Notebook: r570_orthogroupProgenitorAnalysis_forSupp.ipynb).

#### Structural variants

To identify the large structural rearrangements (inversions, translocations and inverted translocations) and local variations (insertions and deletions), each homeologous chromosome group (B, C, D, E, F, G) was aligned to chromosome A using minimap2 (v.2.20-r1061)^57^ with parameter setting ‘-ax asm5 -eqx’. The resulting alignments were used to identify structural variations with SyRI (v.1.6)^71^ and annotation gff3 was used to obtain genes affected by variations between homeologous chromosomes.

#### Orthogroup diversity

Calculation of mean pairwise differences among progenitor specific homeologs was performed by first extracting all pairwise combinations of progenitor assigned alleles within orthogroups that were anchored by an *S. bicolor* ortholog. Among these, 25,000 peptide pairs per progenitor were randomly selected and pairwise aligned using R package Biostrings (v.2.70.2)^72^. Pairwise identity calculation was based on matches/alignment length (PID2; script PID_calc.R). Multiple sequence alignments among syntenic orthogroups for sugar transport gene candidates were performed using MAFFT (v.7.487)^73^ and were visualized using ggmsa^74^ (script MSAalignmentPlots.R). Fold scores for each peptide were calculated using ESMfold (v.2.0.1)^75^.

#### Resistance gene analogues

RGAs were annotated on scaffolds larger than 10 megabases with NLR-Annotator (v.2)^38^ using default parameters. The 4,116 predicted RGAs (Supplementary Table 11) were assigned to progenitors by intersecting the location of each motif with progenitor assignment blocks (Supplementary Table 6).

#### Progenitor divergence

To determine the neutral substitution rate between *S. officinarum* and *S. spontaneum*, 45,000 random ortholog pairs were extracted from all pairwise combinations of progenitor assigned alleles (*n* = 193,815) within *S. bicolor* anchored orthogroups. Peptide sequence pairs were aligned using MAFFT (v.7.487)^73^ and converted into coding sequence (CDS) using pal2nal (v.13)^76^. Pairwise synonymous mutation rates (Ks) among sequences were calculated using seqinr (v.4.2-16)^77^, finding a single synonymous (ks) mutation peak at 0.012 (Supplementary Fig. 13). Assuming a neutral nuclear mutation rate of 0.383 × 10^−8^ to 0.386 × 10^−8^ (ref. ^78^), *S. officinarum* and *S. spontaneum* diverged approximately 1.55–1.56 million years ago.

#### *Bru1* genetic and physical maps

We developed a map-based cloning approach adapted to the high polyploid context of sugarcane to target the durable major rust resistance gene *Bru1*. Haplotype-specific chromosome walking was performed through fine genetic mapping exploiting 2,383 individuals from self-progenies of R570 and physical mapping exploiting two BAC libraries^44,79^. The high-resolution genetic map of the targeted region included flanking markers for *Bru1* (at 0.14 and 0.28 cM), 13 co-segregating markers and the partial BAC physical map of the target haplotype included two gaps^44^; Fig. 3b. To complete the physical map of the target *Bru1* haplotype, we constructed a new BAC library (using enzyme BamHI) using a mix of DNA from four brown-rust-resistant individuals from the R570 S1 population. The BAC library contained 119,040 clones with an average insert size of 130 kb and covered 3.2-fold the target haplotype and 1.6-fold the total genome.

BAC-ends and BAC subclones from the four BACs (CIR009O20, 022M06, CIR012E03 and 164H22) surrounding the two remaining gaps (‘left’ and ‘right’) in the physical map of the *Bru1* haplotype were isolated and used for chromosome walking (as described in ref. ^44^). Two BACs (CIRB251D13 (150 kb) and CIRB286F09 (130 kb)) were identified and sequenced to fill the right gap. Five BACs (CIRB009N07 (100 kb), CIRB114G05 (100 kb), CIRB127D08 (125 kb), CIRB210D07 (105 kb) and CIRB236L05 (150 kb)) reduced the size of the left gap by 35 kb, but an unsized gap remained. The R570 genome assembly spanned the entirety of the *Bru1* target haplotype region with one contig, closing the left gap (99,750 bp) enabling all candidate genes in the region to be investigated (Fig. 3b).

#### *Bru1* candidate genes

The target gap-filled haplotype that represented 0.42 cM and 309 kb was manually annotated, predicting a total of 13 genes (Fig. 3b and Supplementary Table 13). Nine of these genes were also present on all or some of the hom(e)ologous BACs/haplotypes in the R570 genome^27^. Three of the curated genes were present only in the insertion specific to the *Bru1* haplotype. Other whole-genome annotated genes (SoffiXsponR570.03Dg024000; SoffiXsponR570.03Dg024100; SoffiXsponR570.03Dg024600; SoffiXsponR570.03Dg024700) in the region were short, mono-exonic peptides that either contained no protein domains or appeared to be annotated transposable elements, and thus were not supported in the curated candidate gene list (Supplementary Table 13). Among the 13 predicted genes, we searched genes that presented high homology with genes already shown to be involved in resistance mechanisms. We identify five such genes, four genes encoding serine/threonine kinases (genes 1, 5, 7 and 8) and one gene encoding an endoglucanase (gene 13). Annotation of these genes was refined manually through phylogenetic analysis that included genes with high homology from other plants present in databases and search of conserved functional protein domains.

Gene 13, which encodes an endoglucanase, comprised 3 exons and two introns with a genomic size of 1.8 kb for a predicted transcript of 1.5 kb. Sequence alignment and phylogenetic analyses performed with beta-1-4 endoglucanase and beta-1-3 endoglucanase from monocots and dicots showed that gene 13 belongs to the beta-1-4 endoglucanase. This gene presents high homology (greater than 60%) with beta-1-4 endoglucanase from other plants and has the highest homology (88% of identity, 100% coverage) with the orthologous *Miscanthus* gene (CAD6248271.1). Beta-1-4 endoglucanases are involved in cell development^80^ in particular on elongation of the cell wall^81^ but have not been reported as involved in disease resistance. This suggested that this gene is not a good candidate for being *Bru1*.

Gene 1 is composed of eight exons and seven introns. Its genomic size is 4.3 kb and the CDS size is 882 bp. The protein encoded by the gene has 96.5 % identity (100% coverage) with a kinase involved in cell division control in Sorghum (XP_002451427.1) and therefore, it did not appear to be a good candidate.

Gene 5 is composed of six exons and five introns. Its genomic size was 1.1 kb and the predicted CDS size 534 bp. Alignment of its amino acid sequence with Interpro conserved protein domain database showed that only part of the protein (exons 4 to 6) has homology with subdomains VIb to XI of the serine/threonine kinases. This serine/threonine kinase was thus not complete, lacking some of the functional subdomains and appeared to be a pseudogene. Therefore, it did not appear to be a good candidate.

Gene 7 is composed of six exons and five introns, and gene 8 has four exons and three introns. Both present homology with receptor-like kinases. Annotation of conserved protein domains showed that gene 7 has all the 12 subdomains of kinases and thus could encode a functional protein, while gene 8 encompasses only part of these sub domains (I to VII) and could correspond to a pseudokinase. The classification with the ITAK database (http://itak.feilab.net/cgi-bin/itak/index.cgi) revealed they both belong to the RLK-PELLE-DSLV family^45^, the same family to which belong the barley stem rust resistance gene (*RPG1* (ref. ^46^)) and the wheat yellow rust resistance gene (*Yr15* (ref. ^47^)) shown to be a tandem kinase-pseudokinase (TKP). In addition, the third intron of gene 7 has a very large size of approximately 11 kb, including a large TE, a particular structure shared with *RPG1* and *Yr15* TKPs. *Bru1*, like *RPG1* and *Yr15*, is among the relatively rare resistance genes that confer durable fungal resistance. This tandem kinase-pseudokinase (TKP7 and TKP8) is therefore a solid candidate for *Bru1*.

### Reporting summary

Further information on research design is available in the Nature Portfolio Reporting Summary linked to this article.

## Online content

Any methods, additional references, Nature Portfolio reporting summaries, source data, extended data, supplementary information, acknowledgements, peer review information; details of author contributions and competing interests; and statements of data and code availability are available at 10.1038/s41586-024-07231-4.

### Supplementary information

Supplementary Information This file contains Supplementary text and data, Table 1, Figs. 1–15 and References.

Reporting Summary

Supplementary Table 2 Haplotype depth summary across the genome assembly.

Supplementary Table 3 Syntenic orthogroups among *S.bicolor*, *S. spontaneum*, R570 monoploid path, R570 primary assembly.

Supplementary Table 4 Synonymous (Ks) peak among orthologs between *S. officinarum* and *S. spontaneum*.

Supplementary Table 5 Progenitor base assignment summary.

Supplementary Table 6 Progenitor assigned blocks base in genome.

Supplementary Table 7 Haplotype windowed depth blocks across the genome.

Supplementary Table 8 Allelic diversity among progenitors within orthogroups.

Supplementary Table 9 Intersection of genes and haplotype depth.

Supplementary Table 10 List of genes impacted by structural variants.

Supplementary Table 11 Resistance gene analogue motif locations.

Supplementary Table 12 Resistance gene analogue enrichment values.

Supplementary Table 13 Bru1 curated candidate genes and function.

Supplementary Table 14 SRA Bioproject information.

### Source data

Source Data Fig. 2

## Data Availability

Additional work to support the findings of this manuscript can be found in the Supplementary Data section. Sequencing libraries (Illumina DNA/RNA and PacBio continuous long read/HiFi) are publicly available within the sequence read archive (SRA). BioProjects and individual accession numbers are provided in Supplementary Table 14. Genome assembly and annotation for the primary assembly is freely available at Phytozome (https://phytozome-next.jgi.doe.gov/). This Whole Genome Shotgun project has been deposited at DDBJ/ENA/GenBank under the accession JAQSUU000000000. The version described in this paper is JAQSUU010000000. Publicly available genomes used for comparative genomics can be downloaded here: *Setaria viridis* (v.2.1; https://phytozome-next.jgi.doe.gov/info/Sviridis_v2_1), *Sorghum bicolor* (v.3.1; https://phytozome-next.jgi.doe.gov/info/Sbicolor_v3_1_1), R570 monoploid tiling path (http://sugarcane-genome.cirad.fr) and *Saccharum spontaneum* (http://www.life.illinois.edu/ming/downloads/Spontaneum_genome/). Raw data used for analysis in this paper are freely available on figshare (10.6084/m9.figshare.22138004). Source data are provided with this paper.

## Code availability

Scripts and data files used for analysis in this paper are freely available on figshare (10.6084/m9.figshare.22138004) and on GitHub (https://github.com/a-healey/r570scripts).
