# Supplementary material for: The complex polyploid genome architecture of sugarcane
Source: Nature. 2024 Mar 27;628(8009):804–10. doi: 10.1038/s41586-024-07231-4 (PMC11041754; doi:10.1038/s41586-024-07231-4)
Supplement: Supplementary file 1 — This file contains Supplementary text and data, Table 1, Figs. 1–15 and References. [file 41586_2024_7231_MOESM1_ESM.pdf]

nature portfolio

https://doi.org/10.1038/s41586-024-07231-4

Supplementary information

The complex polyploid genome architecture of sugarcane

In the format provided by the
authors and unedited

Supplementary Data Guide for: The complex polyploid genome architecture of sugarcane

Author Information
AL Healey*[1], O Garsmeur[2,3], JT Lovell[1,4], S Shengquiang[4], A Sreedasyam[1], J Jenkins[1], CB Plott[1], N Piperidis[5], N Pompidor[2,3], V Llaca[6], CJ Metcalfe[7], J Doležel[8], P Cápal[8], JW Carlson[4], JY Hoarau[2,3,9], C Hervouet[2,3], C Zini[2,3], A Dievart[2,3], A Lipzen[4], M Williams[1], LB Boston[1], J Webber[1], K Keymanesh[4], S Tejomurthula[4], S Rajasekar[10], R Suchecki[11], A Furtado[12], G May[6], P Parakkal[6], BA Simmons[12,13], K Barry[4], RJ Henry[12,14], J Grimwood[1], KS Aitken[7], J Schmutz*[1,4], A D'Hont*[2,3]

1-Genome Sequencing Center, HudsonAlpha Institute for Biotechnology, Huntsville, AL, USA
2-CIRAD, UMR AGAP Institut, F-34398 Montpellier, France
3-UMR AGAP Institut, Univ Montpellier, CIRAD, INRAE, Institut Agro, Montpellier, France
4-Department of Energy Joint Genome Institute, Lawrence Berkeley National Laboratory, Berkeley, CA, USA
5-Sugar Research Australia, 26135 Peak Downs Highway, Te Kowai, Qld 4741, Australia
6-Corteva Agriscience, Johnston IA, USA
7-CSIRO Agriculture and Food, Queensland Bioscience Precinct, St Lucia, QLD, Australia
8-Institute of Experimental Botany of the Czech Academy of Sciences, Centre of Plant Structural and Functional Genomics, CZ-779 00 Olomouc, Czech Republic
9-ERCANE, 29 rue d'Emmerez de Charmoy, 97490 Sainte-Clotilde, La Réunion, France
10-Arizona Genomics Institute, University of Arizona, Tucson, AZ, USA
11-CSIRO Agriculture and Food, Urrbrae, SA 5064, Australia
12-Queensland Alliance for Agriculture and Food Innovation, University of Queensland, Brisbane, QLD, Australia
13- Joint BioEnergy Institute, Lawrence Berkeley National Laboratory, Emeryville CA, USA
14-ARC Centre of Excellence for Plant Success in Nature and Agriculture, University of Queensland, Brisbane, QLD, Australia

*Corresponding authors

Table Of Contents

Introduction and rationale	Page 1
Simplex marker generation and genetic map construction	Page 2-3
Single flow-sorted library sequencing and marker generation	Page 2-5
Simplex marker clustering and initial chromosome construction	Page 5-8
Single chromosome library clustering and joins	Page 8-9
Misjoin identification	Page 9-11
Sorghum bicolor synteny joins and HiC inspection	Page 11-12
R570 alternate assembly rationale and inspection	Page 12-16
Progenitor kmer generation and block calls	Page 16-17
Final chromosome inspection	Page 17

SUPPLEMENTAL FIGURES

Supplemental Figure 1	Sliding window genetic marker extraction example	Page 3
Supplemental Figure 2	Transcript coverage plot for single flow-sorted chromosome libraries	Page 5
Supplemental Figure 3	Simplex marker barplots for scaffold clustering	Page 6
Supplemental Figure 4	Simplex marker chromosome clustering and initial chromosome construction	Page 8
Supplementary Figure 5	Single chromosome marker joins	Page 9
Supplementary Figure 6	Misjoin identification using simplex and single chromosome library markers	Page 10
Supplementary Figure 7	Tertiary chromosome joins using Sorghum gene synteny and HiC	Page 11
Supplementary Figure 8	Overlap trimming among contigs for chromosome construction	Page 12
Supplementary Figure 9	PacBio HiFi split haplotypes	Page 14
Supplementary Figure 10	Chromosome/contig similarity comparisons	Page 15
Supplementary Figure 11	Progenitor assignments using species specific kmers	Page 17
Supplementary Figure 12	Gene/repeat density within the primary genome assembly	Page 20
Supplemental Figure 13	Synonymous (Ks) peak among ortholog sequences between *S. officinarum* and *S. spontaneum*.	Page 21
Supplementary Figure 14	Haplotype/progenitor assignments in the primary assembly	Page 22
Supplementary Figure 15	Single chromosome sort flow cytometry example	Page 23

SUPPLEMENTAL TABLES

Supplemental Table 1	R570 Genome Resource Overview	Page 1
Supplemental Table 2	Haplotype depth summary across the genome assembly	
Supplemental Table 3	Syntenic orthogroups among *S.bicolor*, *S. spontaneum*, R570 monoploid path, R570 primary assembly	
Supplemental Table 4	Synonymous (Ks) peak among orthologs between *S. officinarum* and *S. spontaneum*.	
Supplemental Table 5	Progenitor base assignment summary	
Supplemental Table 6	Progenitor assigned blocks base in genome	
Supplemental Table 7	Haplotype windowed depth blocks across the genome	
Supplemental Table 8	Allelic diversity among progenitors within orthogroups	
Supplemental Table 9	Intersection of genes and haplotype depth	
Supplemental Table 10	List of genes impacted by structural variants	
Supplemental Table 11	Resistance gene analog motif locations	
Supplemental Table 12	Resistance gene analog enrichment values	
Supplemental Table 13	Bru1 curated candidate genes and function	
Supplemental Table 14	SRA Bioproject information	

Supplementary Data

The biology and pedigree of R570 poses significant technical challenges during genome assembly, the largest being the task of constructing biologically representative chromosomes for an interspecific hybrid crop with variable ploidy, heterozygosity and redundancy introduced through backcrossing and 2n+n chromosome transmission. Equally challenging was accurately representing a partially redundant genome with varying numbers of chromosomes that each do not always pair with the same homeologous copy (polysomic inheritance). While chromosome construction for the majority of plant genomes can be completed using an optical map or Hi-C scaffolding, the unique biology and breeding of R570 (discussed in the main manuscript) requires additional genomic resources (eg. simplex markers, single flow sorted chromosome libraries, Hi-C, optical map, *Sorghum* gene synteny, progenitor specific kmers) in order to separate, phase and construct homeologous chromosomes. Below is an outline of each genomic resource constructed and its function/rationale in the genome assembly process. Our intent with this document is to showcase the strengths and weaknesses of each genome resource/technology so that other researchers can avoid pitfalls and the assumption that a single bioinformatics pipeline/strategy will work for other highly complex genomes. The overall assembly pipeline required the generation and inspection of thousands of plots (each of which was re-generated when new joins/breaks/rearrangements were investigated) used to order and orient contigs into homeologous chromosomes. To help illustrate how the assembly was completed, scripts, commands and representative figures are provided to help guide others through each step of the process.

Supplemental Table 1- R570 Genome Resource Overview. Listed below are each of the genomic sequencing technologies and techniques used to construct chromosomes for sugarcane R570, along with how they were generated and what their function when scaffolding contigs into chromosomes. Please note that this table includes only resources used for assembling the genome. Other resources (ie. RNAseq, IsoSeq) and analyses (eg. HiFi reads- inference of haplotype collapse) are described in the main text.

**Resource**	**Number/Sequencing Coverage**	**Tissue source**	**Functions**
Illumina short reads	~80x per haplotype	R570 leaf tissue	Construction of genetic markers for genotyping; assembly polishing
PacBio continuous long reads	~159x per haplotype	R570 leaf tissue	Assembled into contigs used for draft chromosome construction (genome assembly version 1)
PacBio HiFi reads	~38X per haplotype	R570 leaf tissue	Assembled into contigs/chromosomes for genome assembly version 2.
Selfed (‘S1’) R570	~15x coverage Illumina	96 selfed offspring leaf	Testing marker

offspring	2x150 PE reads	tissue	segregation for simplex inheritance (3:1 presence: absence)
Simplex markers	n=1,825,092	Developed from 3:1 segregation S1 progeny	Genetic map construction. Clustering contigs from homeologous chromosomes for joins. Quality control for new joins.
Single chromosome sort libraries (SCL markers)	N=79 libraries; ~115X per library; 500,092 markers	Single, flow-sorted R570 chromosomes, sequenced with Illumina, assembled into contigs	Clustering contigs for joins with uniquely mapping markers. Quality control for new joins
Optical Map	101X	Bionano Optical Map generated by Corteva Agriscience	Initial long-range scaffolding
Hi-C	~56X	R570 leaf tissue	Quality control for joins
Sorghum bicolor primary proteins	n=34,129 primary peptides	Reference genome annotation (v3.1)- freely available on Phytozome	Finding overlap breakpoints between joined contigs based on alignment copy number counts. Initial ordering and orientation for clustered contigs (simplex and SCL)

Simplex markers and the genetic map

The selfed offspring population (S1) of R570 represents a unique resource for contig phasing and chromosome construction. Segregating populations like S1, which are often used for quantitative trait locus mapping, can also enable phased chromosome construction in complex polyploids. Simplex, or single dose, markers are regions of the genome where a single variant distinguishes one locus among all homeologous chromosomes (octoploid genotype example: CTTTTTTT). Simplex markers, with dominant inheritance, segregate at a 3:1 ratio in selfed progeny regardless of ploidy number or chromosome pairing[1], and therefore control for the potential confounding effects of similar sequences among homologous but non-recombining chromosomes. This is ideal for R570 considering its high ploidy and differential chromosome pairing affinity during meiosis.

To generate simplex markers for chromosome construction and phasing, we first constructed a genome assembly from Illumina-only data. Approximately 80X per haplotype genome coverage (~1.9 terabases) was assembled using HipMer [2], (parameters: -k 101), generating 1,735,266 contigs (Total size: 5.03 Gb;

Scaffold N50: 56.1 Kb). To generate genetic markers with anchored physical locations across the genome, the Illumina read libraries were aligned back to the genome assembly using BWA-MEM (version 0.7.5) [3]. Using custom Python scripts (bedFileFromFai.py; extractMarkersFromBam.py), genetic markers were extracted from the resulting bam alignment file by sliding a 80bp non-overlapping window across each alignment position and collapsing and counting all 80bp kmer present from reads which aligned with a primary mapping quality of 50 or greater (Supplementary Figure 1). This approach generated 55,875,929 putative genetic markers across 38,717,727 windows (hereby referred to as the Full Marker Set; or FMS).

```
Scaffold33:26973-27053 ATCTTGGAATAACTTTGTTTGCTCATCCATCAATTCTCTTGCAATTTGCTTGGCAAGCTCGGCAGCATCAGCCTGCGTCT REF
Scaffold33:26973-27053 ATCTTGGAATAACTTTGTTTGCTCATCCATCAATTCTCTTGCAATTTGCTTGGCAAGCTCGGCAGCATCAGCCTGCGTCT 100
=====================================
Scaffold33:27054-27134 TTCTGTGTCTGACATTATTTCTCCTCACGTGGTGAAGCGCTATGTATGAGGACCTTGCTTTGATACCAAATGAAAGAATA REF
Scaffold33:27054-27134 TTCTGTGTCTGACATTATTTCTCCTCACGTGGTGAAGCGCTATGTATGAGGACCTTGCTTTGATACCAAATGAAAGAATA 77
Scaffold33:27054-27134 TTCTGTGTCTGACATTATTTCTCCTCACGTGGTGAAGCGCTATGTATGAGGACCTTGCTTTGATACCAATTGAAAGAATA 11
=====================================
Scaffold33:27135-27215 TATAATGCCTAAAGGGGGTCAATAGGCGCATCTAAAAATTTTACAACACAAACTCAAGTTCAATGTCAGCAACTGCCGGA REF
Scaffold33:27135-27215 TATAATGCCTAAAGGGGGTCAATAGGCGCATCTAAAAATTTTACAACACAAACTCAAGTTCAATGTCAGCAACTGCCGGA 96
=====================================
Scaffold33:27216-27296 ACCTGACAGTTTAGGCTAGAAATTCTGATGGTCAAAAGTTCTGACGCAAACTGGCTGGCAGTTCCGACTCCTATGTGAAC REF
Scaffold33:27216-27296 ACCTGACAGTTTAGGCTAGAAATTCTGATGGTCAAAAGTTCTGACGCAAACTGGCTGGCAGTTCCGACTCCTATGTGAAC 96
=====================================
Scaffold33:27297-27377 ATGAAATCAGTGACAAAATCAACTTTGAGTATGAGTTTGCTTACAACCCCACTTCCTAGTGGTTAAGTAAAGATGTAGGA REF
=====================================
Scaffold33:27378-27458 CACTCCCTTGACGTCGAACGGCCTCCACTCCGTAGATTAGGTTCCAACCCTAGGAAAGAGCTAAAGGGAGAAAGAGACAA REF
Scaffold33:27378-27458 CACTCCCTTGACGTCGAACGGCCTCCACTCCGTAGATTAGGTTCCAACCCTAGGAAAGAGCTAAAGGGAGAAAGAGACAA 26
=====================================
Scaffold33:27459-27539 CAAGAATAAGTGATTACAAACAATTCACAACAACAAGCACACGAAGACAATGATTTATATATCCTGAGGTTCGGAAACCC REF
Scaffold33:27459-27539 CAAGAATAAGTGATTACAAACAATTCACAACAACAAGCACACGAAGACAATGATTTATATATCCTGAGGTTCGGAAACCC 40
=====================================
Scaffold33:27540-27620 ACAAAGGAGCTCCTACGTCCTCGTTGTTAAGGTGACCACTAAGGTCAGAGTCTCTTCCACCTCCTTGTCTCTCTCAAGGA REF
Scaffold33:27540-27620 ACAAAGGAGCTCCTACGTCCTCGTTGTTAAGGTGACCACTAAGGTCAGAGTCTCTTCCACCTCCTTGTCTCTCTCAAGGA 6
=====================================
Scaffold33:27621-27701 NO ALIGNMENTS
=====================================
Scaffold33:27702-27782 NO ALIGNMENTS
```

Supplementary Figure 1- Example output of the sliding window marker extraction. Non-overlapping 80bp windows were extracted from the alignment bam file where Illumina reads (approximately 80X coverage) were aligned to HipMer scaffolds. The reference sequence per window was printed first (REF line), followed by all possible kmers from Illumina reads that aligned fully across the window with mapping qualities greater than or equal to 50. If no reads had a mapping quality greater than 50 for a given window, then only the reference sequence was printed. Additionally, if there were no unique alignments across a given window, then no marker or reference sequence was generated (NO ALIGNMENTS).

To determine which markers were simplex and could be used to generate a genetic map and phase contigs, the FMS were genotyped in 96 selfed (S1) offspring from R570 to identify markers demonstrating the expected 3:1 segregation pattern of simplex markers in an S1 population. Each FMS marker was matched and counted within the re-sequenced population of S1 offspring, which were sequenced to a depth of 15X. As spurious matches can occur simply due to sequencing error, marker genotype counts greater than one but less than or equal to three were labeled as NA. The remaining markers were coded as zero for absent or one as present. Each marker's segregation pattern was tested using a binomial test ($p < 0.1$), finding 1,825,092 simplex markers.

To ensure a complete marker set for generating a genetic map, missing data within the simplex marker genotype matrix were imputed from 50 marker sliding windows, which reduced the amount of missing marker calls in the matrix from approximately 13.5% to 0.2%. Based on recombination frequency, the marker set was culled down to 10,732 non-identical (redundant) markers that could be used for genetic map construction. Linkage groups from the raw simplex markers were calculated in JoinMap v4.0[4], retaining markers within the same linkage group that had a pairwise LOD score of ≥ 3 and a recombination fraction ≤ 0.05. These parameters produced 237 tightly-linked linkage groups that were unlikely to include spurious joins. Marker order was inferred within linkage groups via a four step pipeline: (1) missing data were k-nearest neighbor imputed using the median method in DMwR (v0.4.1) [5]; (2) recombination fractions were estimated in R/qtl (v1.42-8) [6]; (3) the resulting matrix was fed into tspMap [7] and markers were ordered using the concorde algorithm; (4) genetic map estimation was accomplished in R/qtl using the kosambi mapping function, an error probability of 0.01. With this scaffold of high-confidence marker orders, we then interpolated the positions of all simplex markers, which were then used to cluster contigs with shared linkage for chromosome construction.

Single flow-sorted single chromosome libraries

While the genetic map markers were the useful for determining phase in a complex, high ploidy system such as sugarcane, their presence and detection is skewed toward the more heterozygous, *S. spontaneum* portion of the genome (131 Mb of simplex markers; 45% derived from *S. spontaneum* portions of the genome; Fisher's Exact Test: 3.25x enrichment for simplex markers in *S. spontaneum* regions [p<0.0001]). To generate more genetic markers that distinguish among other homeologous chromosomes through non-simplex regions, flow cytometry was used to sort and sequence single R570 chromosomes (n=79; ~115X coverage) using methodology described in the main text. Each library corresponded to one physical chromosome [8,9], and enabled coarse-grained clustering of contigs into homologous chromosome groups from unique marker placements.

To first distinguish which homologous chromosome was captured per sequencing library, reads were aligned to *Sorghum bicolor* (v3.1)[10] primary transcripts using BWA-MEM (version 0.7.5) [11] to calculate per transcript percent coverage (parameters: bedtools genomecov -bga -ibam)[12]. Coverage per transcript was calculated for each base with five or more reads aligned (intended to ignore differences in sequence divergence between R570 and *S. bicolor* and repeat sequences). Percent coverage per transcript was converted to a scatterplot and visualized (Supplementary Figure 2). This approach enabled quick determination of: 1) which homologous chromosome copy had been flow-sorted, captured and sequenced, 2) whether a recombinant chromosome was captured, or 3) if there was a problem with the library that would result in uninterpretable marker placements/patterns downstream.

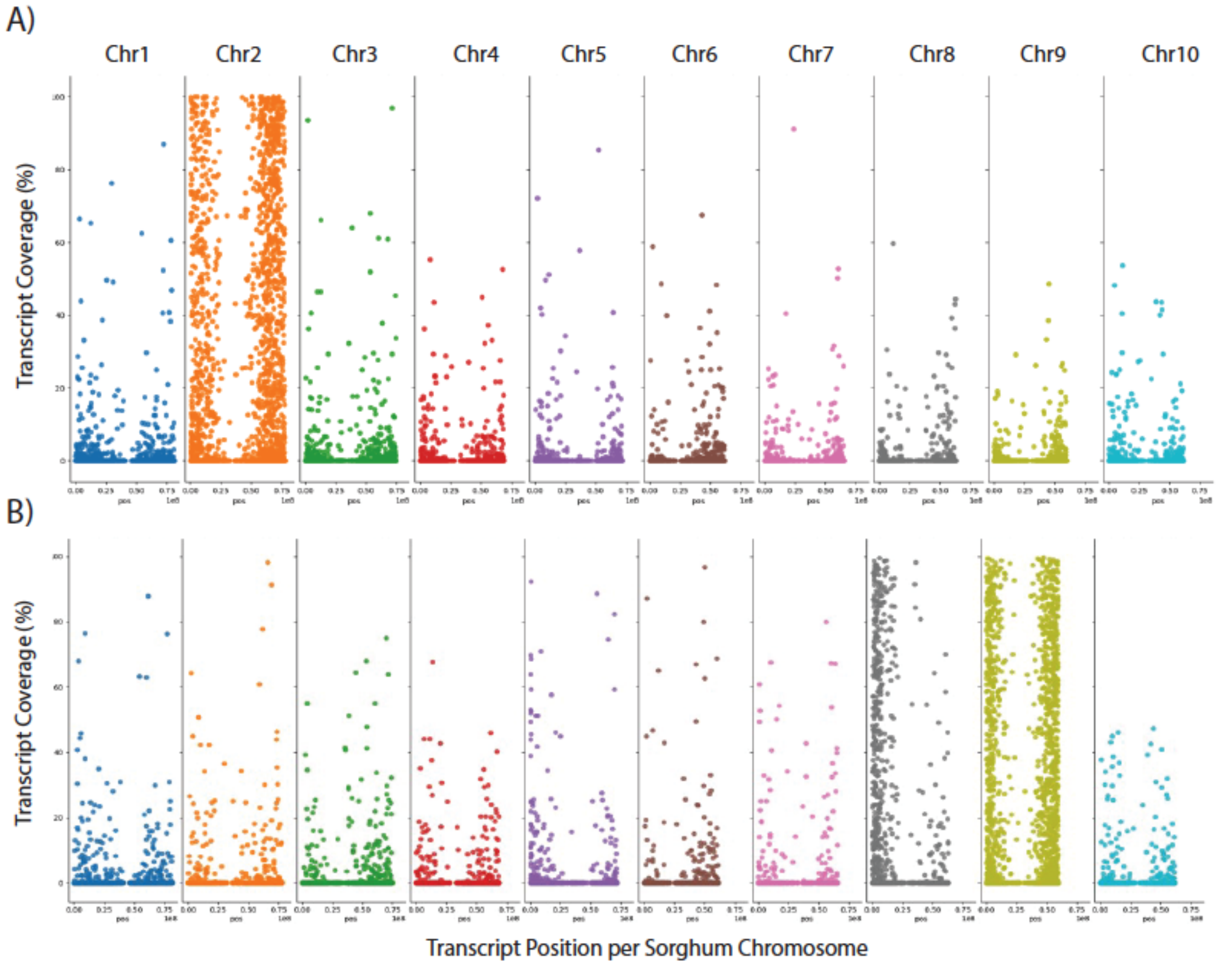

Supplementary Figure 2- *Sorghum bicolor* transcript coverage by single flow-sorted chromosome libraries from R570. Reads from individually flow-sorted and sequenced chromosomes were aligned to *S.bicolor* (v3.1) transcripts to calculate percent coverage to transcript base. The example in panel A) shows a single homologous chromosome for *S.bicolor* chromosome 2 (R570 chromosome 5). Panel B) shows a *S. spontaneum* chromosome (fusion between Sb08 and Sb09; R570- Chr6_9).

Each of the single flow-sorted sequenced chromosome libraries was visually inspected using the approach described above. Chromosome-specific markers for each homeolog were then generated by assembling each flow cytometry-sorted library into contigs using HipMer [2](parameters: -k 101), then extracting 500bp markers within each assembly, spaced every 2000bp from contigs greater than 1 kb (script: extractUniqueMarkersFromFasta.py). This approach generated 500,092 chromosome specific markers from 79 Illumina libraries (SCL marker set). This marker set, along with the simplex markers were used to order and orient contigs into chromosomes (primary and secondary joins).

R570 Chromosome Construction

Contig construction and scaffolding

The R570 contigs were assembled from approximately 38X coverage per haplotype of PacBio HiFi data using HiFiAsm (version 0.13-r308) with the resulting contigs polished with RACON (version 1.4.10)[3]. Comparison to a previous R570 assembly (version 1.0-PacBio consensus long read; ~159X assembled with CANU[13], polished with ARROW[14]) showed a 20X contiguity improvement (484.3 Kb and 10 Mb, respectively), so we proceeded with chromosome construction from HiFi contigs only.

Initial scaffolding of the genome was completed using a Bionano Optical map generated by Corteva (methods described in main document text); however it became apparent that the identical regions of the genome, mainly contributed from *S. officinarum*, caused large segmental duplications in scaffold ordering, as negative gaps are allowed to occur. Additionally, with a large portion of the genome being identical among multiple homeologous chromosomes, an out-of-phase contig could easily be selected during optical map scaffolding. For this reason, the contig order within the optical scaffold agp file was used as an initial guide during chromosome construction but priority was given to alignment of genetic map and single sorted library markers (respectively), discussed below.

To cluster sequences derived from the same homeologous chromosome (primary joins), simplex markers were aligned to optical map scaffolds using parallel BLAT (pblat) (v2.5)[15], retaining only single placement, perfect coverage alignments (parameters: -noHead -extendThroughN -minIdentity=100 -fastMap -minMatch=3 -tileSize=12 -minScore=80). As there was no straightforward way to ascertain the expected number of linkage groups that could be associated with contigs that vary in length, the most straightforward approach was to simply count the number of simplex markers (and their associated linkage group) that aligned to each scaffold, and barplot each for visual inspection. Sequences sharing the same linkage information were then manually clustered together to construct individual chromosomes. For example: optical scaffold_1000034 (Supplementary Figure 3) could only be ordered and oriented with other scaffolds with simplex markers associated with linkage groups 79, 201 and 4.

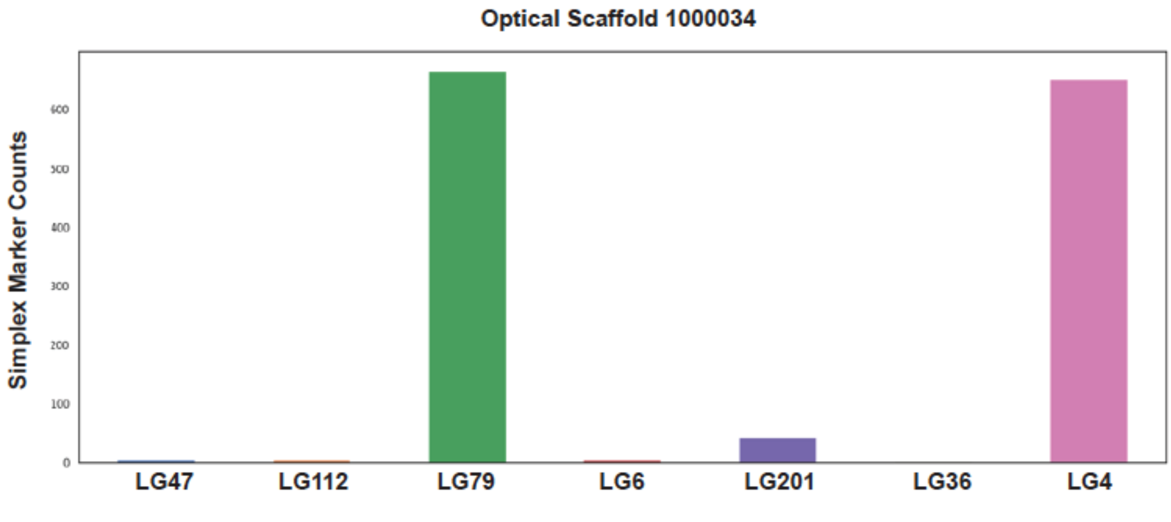

Supplementary Figure 3- Simplex marker barplots for sequence clustering. Simplex markers (with their genetic map associated linkage group [LG]) were aligned to each optical scaffold. Perfect alignments were counted and plotted to record which scaffolds were associated with each linkage group from the R570 genetic map.

To generate a putative join order to each chromosome contig cluster, *S. bicolor* (v3.1) primary peptide sequences were aligned to each cluster using pblat (version v2.5; parameters: -noHead -extendThroughN -tileSize=5 -minIdentity=60 -t=dnax -q=prot -minMatch=2). Contigs and scaffolds with shared linkage were then ordered and oriented relative to *S. bicolor* peptide order. Additionally, using this approach we were able to identify both falsely-joined regions and redundant sequences.

We inspected large-scale overlapping contigs through a screen of gene alignment copy number relative to the *S. bicolor* outgroup. For example, take the case where two contigs [‘A’, ‘B’] could be joined to generate a scaffold. When oriented relative to *S.bicolor* and joined, overlap between contigs A and B can be detected by aligning primary *S. bicolor* peptides and counting the number of high-quality alignments (greater than 85% identity and 80% coverage) that occur. To trim regions of overlap, alignment position and copy number was sorted relative to the shortest contig (in this example: contig A) used to make the join. Breaks were then made at the midpoint position where alignment copy number changed from one to two (example of sorted *S. bicolor* alignment copy number on contig A: 1111111:222222; : = breakpoint; example: Supplementary Figure 4). During the clustering process, there were also instances where *S. bicolor* alignment copy number found redundant sequences that were ignored when making chromosome joins. For example, consider two contigs ['X','Y'] could be joined to an existing scaffold. When contigs X and Y are considered together, all *S. bicolor* alignments counts on contig X are duplicated [example of sorted *S. bicolor* alignment copy number on contig X: 2222222], whereas only some are on contig Y [example of sorted *S. bicolor* alignment copy number on contig Y: 111111222222211111]. In this instance, contig Y would be favored over contig X. All broken sequences and unused, redundant contigs were retained during these steps to ensure no potential coding regions were lost.

We also considered situations where tandem gene duplicates were erroneously removed when determining breakpoints between two adjacent contigs based on alignment copy number, but these would be exceedingly rare. First, an ancestrally single-copy gene family would have had to be duplicated so that two copies exist in one R570 haplotype, while other R570 haplotypes and *S. bicolor* contain exactly one copy; as such, single-copy gene alignments would exist between some, but not all, R570 contigs and S. bicolor. This situation is already rare: 1.2% of all gene families are ≥2-copy in one R570 chromosome and 1-copy in all other haplotypes and *S. bicolor*). Second, the contig break must have occurred so that exactly one copy of the family is represented on each of two adjacent contigs. If such a rare event had occurred, NucFreq analysis [16] (described in the main manuscript) would have likely uncovered it unless the sequences were perfectly identical between the haplotypes.

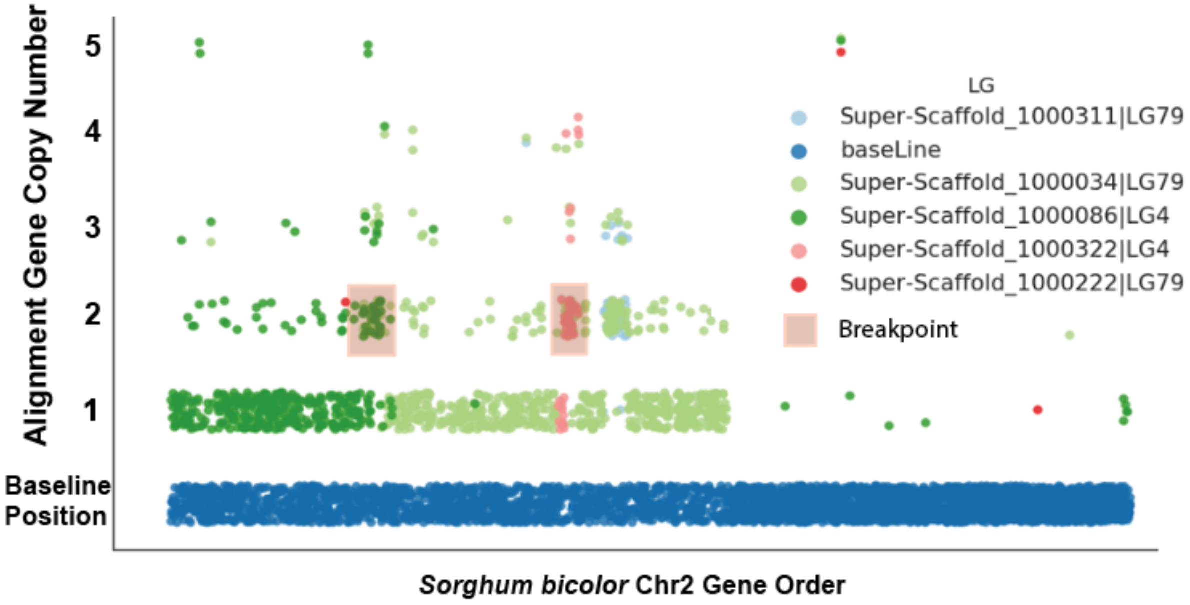

Supplementary Figure 4- Simplex marker chromosome construction. *Sorghum bicolor* proteins are aligned to scaffolds that share simplex marker linkage groups to determine putative joins and breakpoint positions. Each scaffold is listed along with the linkage group with the majority of associated simplex markers. Alignment copy number is used to determine breakpoints between scaffolds, where alignment gene copy number is greater than one between joined, adjacent scaffolds. In this example, two scaffold breaks were made. Scaffold_1000311 (light blue) was not included in this constructed chromosome because its inclusion would only duplicate gene copy alignments (contig X example from previous paragraph). Baseline position for each peptide sequence was provided by relative order within the *S.bicolor* (v3.1) annotation.

Secondary joins were made among linkage cluster joins by aligning SCL markers to all joined linkage clusters from the same homologous chromosome (eg. *S. bicolor* chromosome 2). Single flow-sorted markers enable joins among scaffolds and contigs where linkage group information was lacking, but could be linked by sequence alignment. Markers from each individual flow-sorted chromosome (script: extractUniqueMarkersFromFasta.py; modified slightly to retain non-unique sequences) were aligned using megablast [17] (parameters: -m 8 -p 95 -a 10 -W 100) to each joined linkage cluster and remaining scaffolds to search for additional joins based on visual inspection of high-density marker hits. Once additional joins were manually identified (Supplementary Figure 5), the sequences were ordered and oriented again based on *S. bicolor* protein alignments, as described above (Supplementary Figure 4). This process was completed in an iterative fashion, where joins were repeatedly made, broken and shuffled to generate the longest path of single copy *S. bicolor* protein alignments through each constructed chromosome.

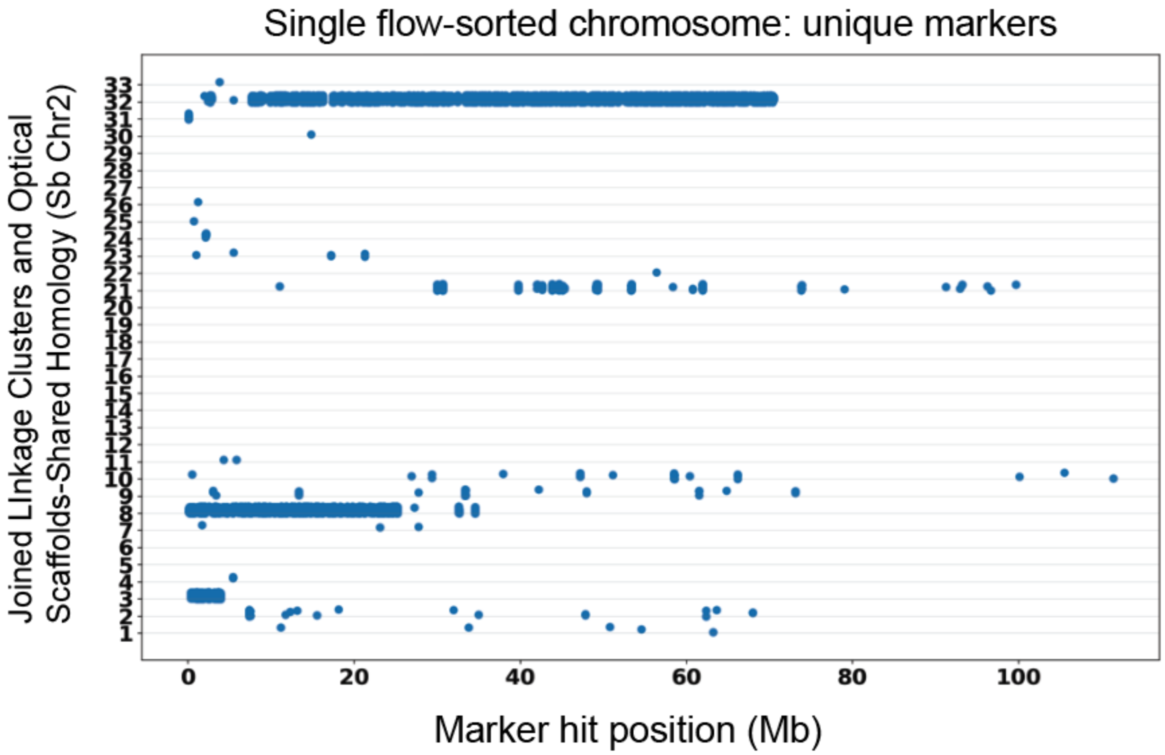

Supplementary Figure 5- Single chromosome marker joins. Genetic markers from each individual flow-sorted chromosome assemblies (SCL markers- previously described) were aligned to all sequences sharing homology. Sequences with high density marker alignments were extracted and inspected further to assess additional joins for constructing chromosomes. In this instance, scaffolds 3,8 and 32 (separate Y-axis scaffold key not provided) were considered for additional joins.

After initial chromosome construction, simplex markers and SCL markers were aligned to each for inspection of possible misjoins (Supplementary Figure 6). If a misjoin was identified, the scaffold was broken, and the placement of the two pieces was re-evaluated. In instances of unclear evidence of a misjoin (example: SCL markers suggest a misjoin but simplex markers do not (and vice versa), multiple lines of evidence were considered together, acting upon the majority consensus. Again, this was completed in an iterative manner until the best possible sequence combinations were confirmed, which required the manual inspection of thousands of plots. Once no further joins could be made based using single chromosome markers, *S.bicolor* proteins were aligned again using pblat[15] (v2.5; previous peptide alignment parameters) to all sequences to inspect whether obvious gaps existed between sequences that, if joined, would generate a more complete assembly (Supplementary Figure 7). These tertiary joins were then inspected by aligning Hi-C to the entire assembly, along with simplex and single chromosome markers, and manually checking whether a misjoin was created or if there was good supporting evidence for the join to be maintained. Hi-C Illumina reads were aligned to the genome assembly using BWA-MEM (version 0.7.5) [11]. Paired-end reads are mapped independently (as single-ends) due to the nature of the Hi-C pair which captures conformation via proximity-ligated fragments. A small fraction of single-end mapped reads will contain a ligation junction. This indicates that they do not originate from a contiguous piece of DNA and are chimeric. In these cases, only the 5'-side was retained since the 3'-end

generally originates from the same contiguous DNA as the 5'-side of the mated read. The resulting single end alignments were combined into a BAM file that contains the paired, 5'-filtered Hi-C read alignments. The 3D-DNA[18] suite of internal tools was used to generate a contact map using the resultant BAM file, and the contact map was visualized using Juicebox (v 1.11.08) [19]. Once all iterative joins were confidently completed, *S.bicolor* peptides were realigned to the assembly to inspect and trim overlaps among scaffolds (Supplementary Figure 8).

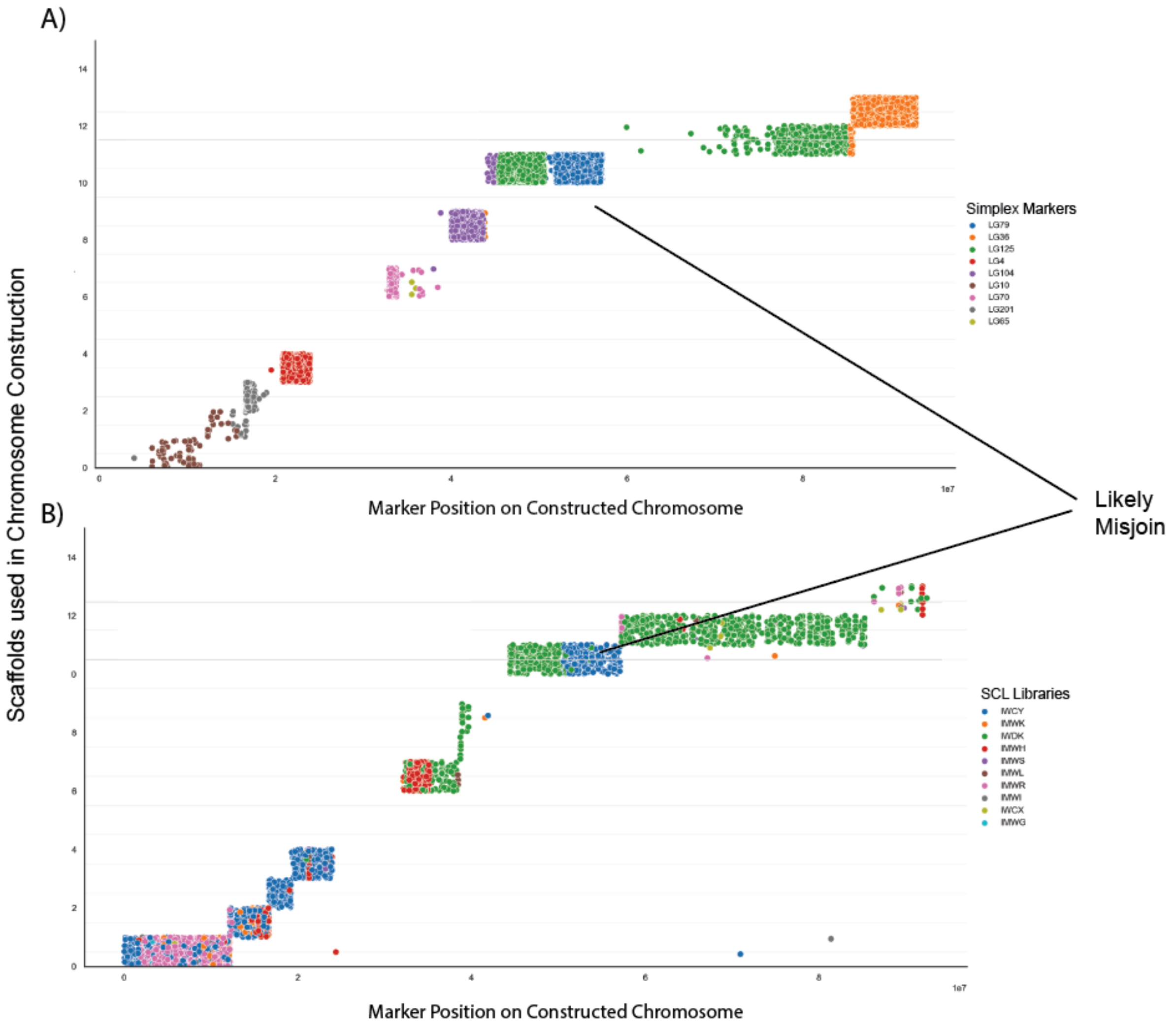

Supplementary Figure 6- Identifying misjoins in the assembly. Once primary (simplex) and secondary (SCL) joins were made, genetic markers were re-aligned to the genome to inspect for misjoins. A) Simplex genetic marker best alignment locations. Each point is colored in based on its correlated linkage group. HiFi scaffolds used in the chromosome construction are separated in each plot on the y-axis to improve visualization (separate Y-axis scaffold key not provided). B) Single chromosome marker best alignment locations. Each alignment point is colored by the Illumina library it was generated from. Each panel is colored independent of another. Scaffold 11 appears to contain a misjoin as both the simplex and SCL markers show an abrupt change to a different linkage group (panel A)/chromosome library (panel B) that reverts back and is not supported on scaffold 12. In this instance, scaffold 11 is broken into two

sequences, the 5' end remains in place while the 3' end is considered for a separate chromosome. Each time a misjoin is identified and corrected, plots (Supplementary Figures 4-6) are re-generated to ensure the new join is supported.

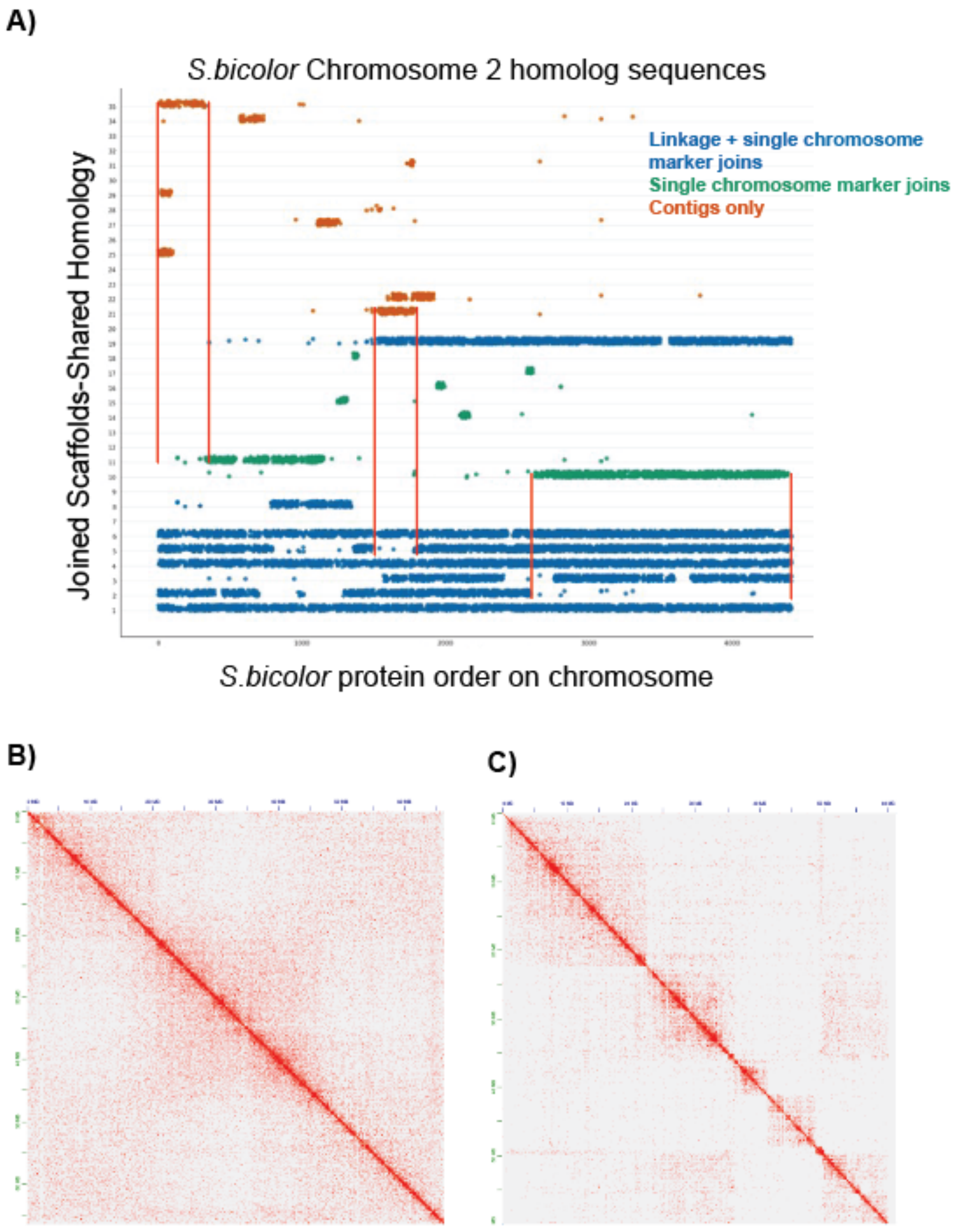

Supplementary Figure 7- Tertiary chromosome joins. Once simplex and single chromosome marker joins are completed, *S. bicolor* peptide sequences are aligned to scaffolds with shared homology, searching for visual gaps that could be filled by other remaining sequences. A) Alignment of *S.bicolor* peptides against all chromosome 2 homologous scaffolds. Scaffolds are colored based on the sequencing

technology/technique that supported the contig joins. X-axis- *S.bicolor* peptide alignments, ordered by chromosome position in *S.bicolor* chromosome 2. Y-axis- all R570 scaffolds with shared homology. In this example, three additional joins were made (indicated by red lines) and inspected with Hi-C. B) Example of well-supported tertiary contig joins. Hi-C contact map shows good, consistent read mappings all along the constructed chromosome. No additional modifications needed. C) Example of tertiary joins that need additional assessment/inspection. Hi-C contact map suggests either gaps or misjoins are present in this assembly.

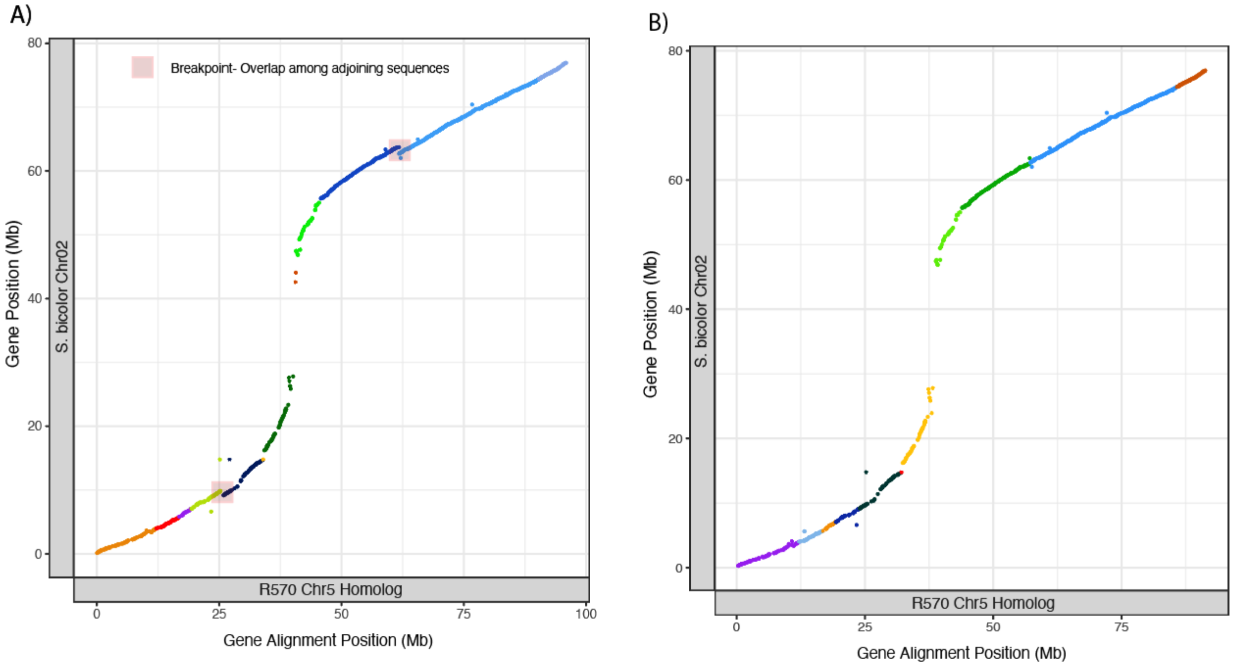

Supplementary Figure 8-Final overlap trimming. Once all joins were completed, *S. bicolor* peptide sequences are aligned to sequences with shared homology to search for sequence overlaps. Overlaps among joined sequences for each individual chromosome are trimmed to maintain long runs of single count gene alignments, leaving the longest contig intact. Panel A shows a tertiary join prior to trimming, while panel B shows the final constructed chromosome (post overlap trimming) with each contig sequence individually colored (independently) to show the overall structure of the chromosome. Trimmed sequences are maintained within the alternate assembly (discussed below).

R570 Alternate Assembly

During the chromosome construction process, the difference between the PacBio CLR (v1.0) and HiFi (v2.0) genome assemblies became apparent, with HiFi assembly splitting apart a larger number of haplotypes in R570. Inspection of marker order from the previous PacBio CLR assembly was performed by extracting 1kb sequences every 2kb from the V1.0 assembly (script: extractUniqueMarkersFromFasta.py; n=1,542,392) which were aligned to HiFi contigs using pblat [15](version 2.5; parameters:-noHead -extendThroughN -minIdentity=99 -fastMap -minMatch=3 -tileSize=12 -minScore=1000). Perfect marker alignments were then visualized (example: Supplementary Figure 9). Alignment of CLR genetic markers on HiFi contigs found many regions of the genome with two perfect paths, where marker positions weaved between separate, but nearly identical contigs.

Assuming these regions were munged together in the previous CLR assembly and are now separated in the HiFi assembly, representing these regions creates two issues with the assembly.

1) As discussed in the main text and represented in the pedigree (Figure 1B), backcrossing, small breeding population sizes, '2n+n' chromosome transmission, and shared maternal/paternal grandparents, results in large regions of the genome that are inbred and highly homozygous. This breeding scheme generates regions of the genome that are impossible to represent in multiple homeologous copies, as one contig represents multiple regions of the genome. Placement of these contigs invariably generates large, megabase gaps elsewhere in the assembly.
2) As these genomic regions can only be separated and distinguished using PacBio HiFi reads (~17Kb), these contigs represent genomic regions that are nearly identical and can only be separated using the latest PacBio long-read technology. Given that downstream analyses of the genome (population genomics, variant detection, etc..) will likely depend on Illumina short read (~150bp) data, including both contigs representing these regions in the same assembly will result large portions of the genome will not contain uniquely mapping reads. This would result in a genome assembly that is less effective for downstream analysis and comparative genomics among R570 and other sugarcane cultivars.

Based on the pattern of possible haplotypes split using HiFi data (example: Supplementary Figure 9), we devised a strategy to manually inspect similarly between sequences. Rather than compare the percent identity across the entire alignment length of two sequences (which only provides a global average and ignores long stretches of near perfect identity), instead we constructed 1kb non-overlapping markers (script: extractUniqueMarkersFromFasta.py) from each iteration of constructed chromosomes, and aligned them to all remaining contigs using pblat[15] **(**v.2.5; parameters:-noHead -extendThroughN -minIdentity=99 -fastMap -minMatch=3 -tileSize=12**)** . If any single marker aligned to a contig with ~99.5% identity, then the contig and the target chromosome (where the marker was derived) were pairwise aligned using nucmer [20] (parameters: -l 100 --maxmatch -b 400) and visually inspected (example plot: Supplementary Figure 10; script: parseMummerToPlot.py). This was iterative, and we re-assessed the alignments after each chromosome build. If contigs had no highly similar match and did not contain any information that would allow their anchoring or ordering within the primary assembly, we included the contig as unanchored sequence in the primary (example: Supplementary Figure 10C). Thus, contigs with highly similarity matches were retained in the alternate bin.

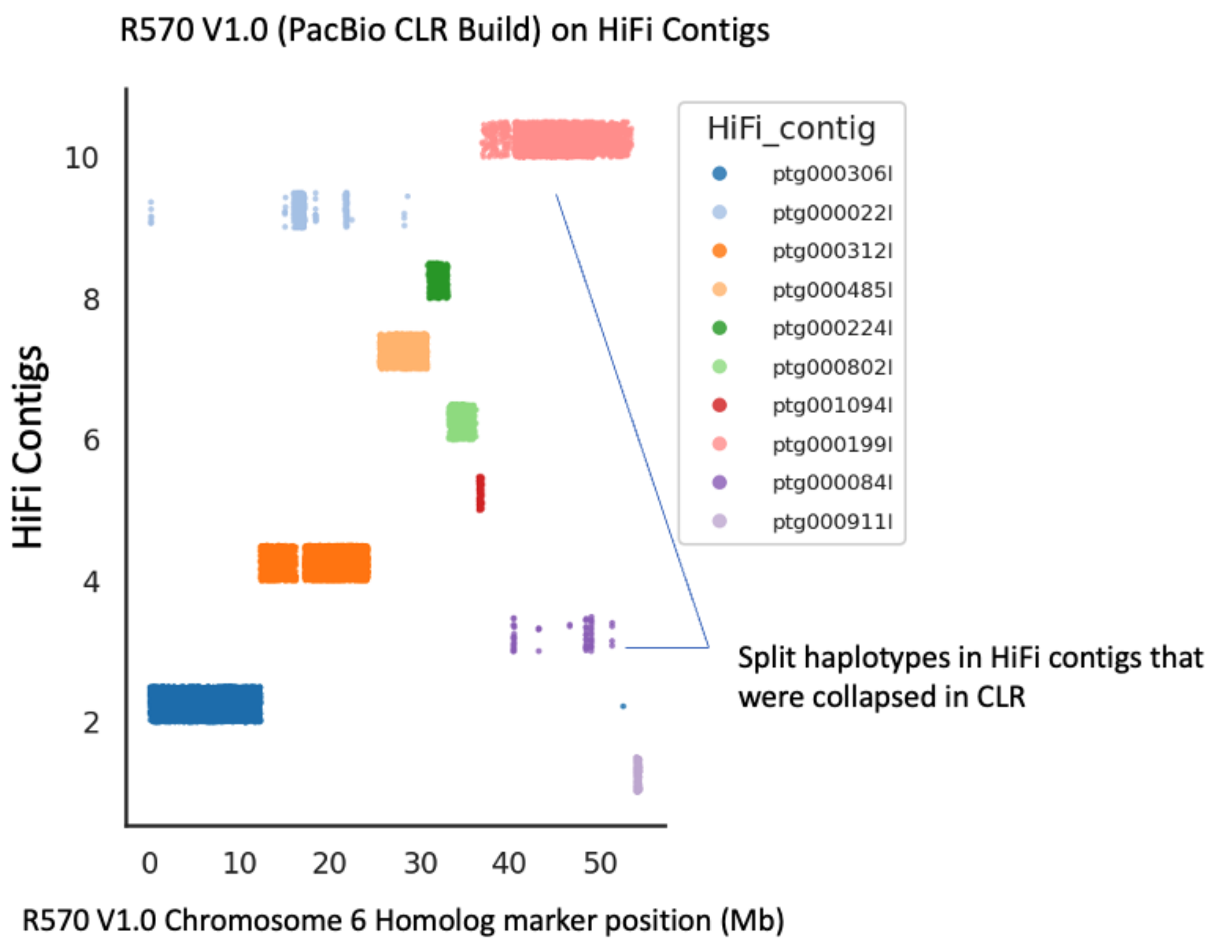

Supplementary Figure 9- PacBio HiFi split haplotypes in R570. Perfect alignment of genetic markers from the V1.0 (CLR) assembly onto HiFi (V2.0) contigs found many regions across the genome where haplotypes could only be split using HiFi reads (example ptg000199l and ptg000084l). Alignment of these regions across the genome found often found sequences that were nearly identical (examples: Extended Data Figure 1C- Chr6E – Chr6E_alt alignment; Supplementary Figure 10- see below) which necessitated the construction and separation of the 'primary' and 'alternate' assembly (discussed above).

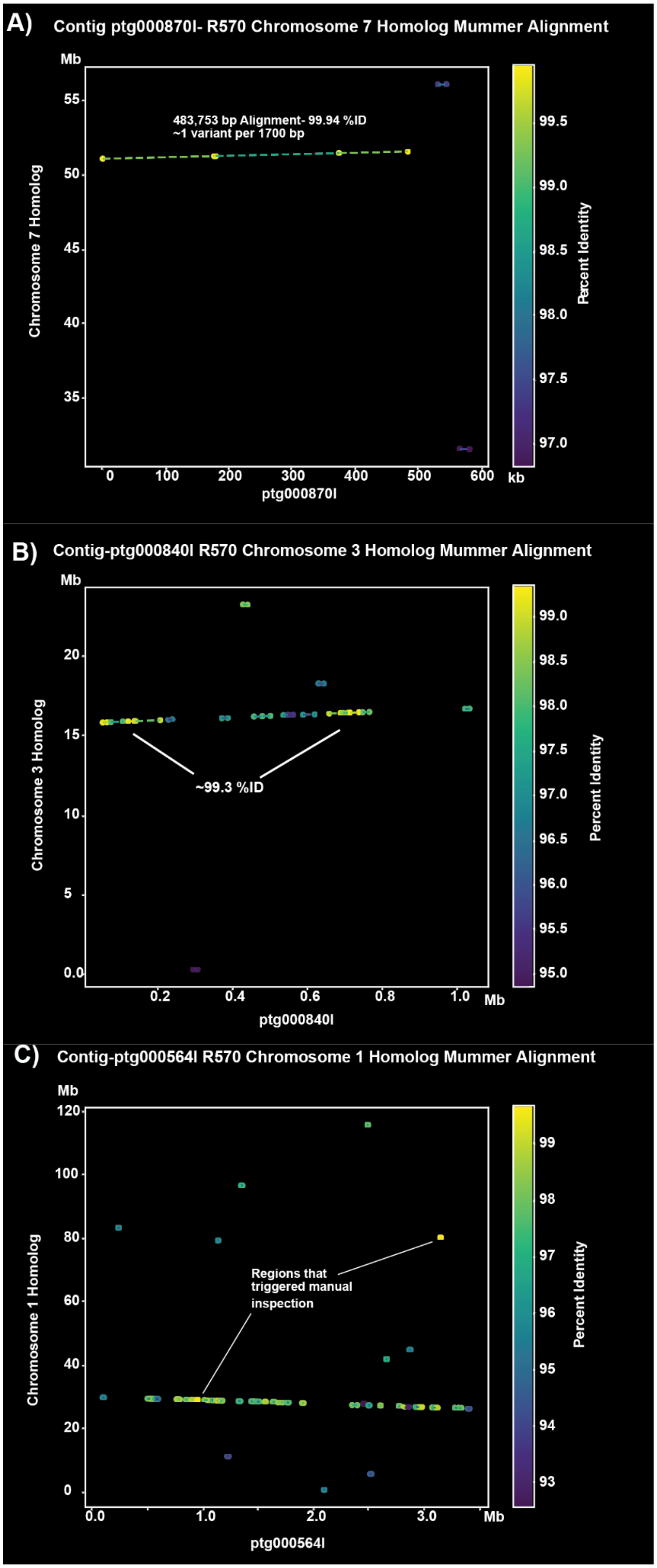
A) Contig ptg000870l- R570 Chromosome 7 Homolog Mummer Alignment
Mb
483,753 bp Alignment- 99.94 %ID
~1 variant per 1700 bp
Chromosome 7 Homolog
ptg000870l
kb
Percent Identity
B) Contig-ptg000840l R570 Chromosome 3 Homolog Mummer Alignment
Mb
~99.3 %ID
Chromosome 3 Homolog
ptg000840l
Percent Identity
C) Contig-ptg000564l R570 Chromosome 1 Homolog Mummer Alignment
Mb
Regions that triggered manual inspection
Chromosome 1 Homolog
ptg000564l
Percent Identity

Supplementary Figure 10- Pairwise Mummer alignments among contigs and constructed chromosomes. One kilobase markers were extracted from each iteration of constructed chromosomes and aligned to all remaining contigs. Any high similarity matches were then visually inspected and manually assessed. For this example, contigs in panels A and B would be placed in the alternate assembly, while the contig in panel C would remain in the primary assembly as an unanchored sequence. Sequences in the primary and alternate assemblies were re-assessed after each version of chromosome construction.

For these reasons outlined above, we opted to bin these sequences separately (primary and alternate, respectively) such that the primary assembly (5.04 Gb) could represent a highly contiguous (12.6 Mb contig N50; 937 contigs), unique representation of the genome with few gaps (0.1%), while the alternate assembly (3.73 Gb) contains the shorter (2.1Mb contig N50; 11,043 contigs), gap prone regions of the genome. To avoid misplacing contigs in the alternative bin that could be used in the primary assembly, chromosome construction was completed in an iterative manner, where contigs were re-assessed for placement elsewhere in the genome assembly, using all genomic resources available. New plots for visual inspection (similar to Supplementary Figures 4-6,10) were generated upon every construction iteration to ensure the best placements of each contig. Once the HiFi primary assembly was finalized, so too was the alternate. When possible, each contig in the alternate assembly was anchored (ordered) against a primary chromosome, in order to improve the downstream utility of the assembly. Please note however, that anchoring and construction of alternate chromosomes does not imply meiotic pairing between primary and alternate chromosomes, and alternate chromosomes can be a mosaic of multiple haplotypes. Anchoring was completed by extracting 5kb, non-overlapping markers (script: extractUniqueMarkersFromFasta.py) from alternate contigs and aligning all to the primary assembly (minimap2 -ax asm5)[21]. Only markers with single, unique placements were considered. In order for an alternate contig to be anchored against a primary assembly sequence, a minimum of four markers from a single contig with at least 90% identity and 98% coverage was required. If these criteria were fulfilled for multiple sequences in the primary assembly, the contig was left unanchored. Of the total sequence in the alternate assembly (3.73 Gb), 1.33Gb was anchored relative to the primary (36%). The alternate assembly is available in the supplemental data and should only be used to query specific allele or gene model questions.

The last step of the chromosome construction pipeline was leveraging microscopy and cytogenetics previously generated to visualize the progenitor contribution in the R570 genome.
To assign progenitor blocks in the genome assembly, 27bp kmers extracted from whole-genome sequencing data from *S. officinarum* (accession LaPurple, SRP159203) and *S. spontaneum* (accession SES234B, SRP159208). In brief, kmers were generated from a subset of 20 million reads from each accession and was considered progenitor specific if it was absent in the other sequencing library at a minimum depth of 10X. Each putative progenitor-specific kmer set was further screened by aligning each to the genome assemblies of *S. spontaneum* [22] and *S. officinarum* (unpublished, NCBI accession number: GCA_020631735.1). Discarding kmers that align to both progenitors, a total of 93,213 *S. officinarum* and 56,351 *S. spontaneum* kmers remained, subsetted to 3,186 and 1,672 (respectively) that provided sufficient exact matches (n= 4,537,931; script: progenitorBlockRLEs.R) to all sequences in the primary and alternate assemblies for progenitor assignment. Exact matches were converted into run-length equivalents using script: progenitorBlockRLEs.R using run lengths of a minimum of 100 consecutive calls from one progenitor (Supplementary Figure 11).

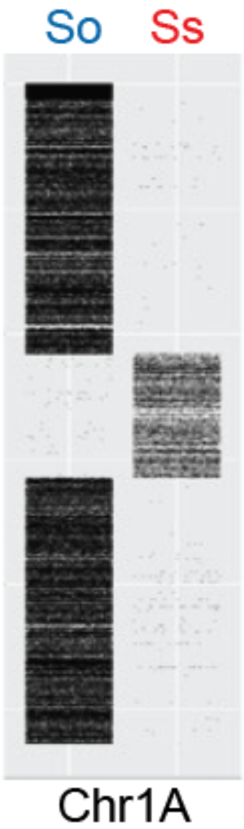

Supplementary Figure 11 - Exact progenitor kmer matches along R570 Chr1A. Matches were converted into progenitor blocks, requiring a minimum of 100 consecutive calls from one progenitor. So- *S.officinarum*; Ss - *S.spontaneum*

The structure of the homoelogous chromosomes were compared to ensure they were consistent with GISH and previous cytogenetic analyses (example: Figure 1C). Lastly, Hi-C data was re-aligned to the primary assembly to ensure the build was consistent and high quality (Extended Data Figure 1B- Hi-C heatmap).

REFERENCES

1. Wu, K. K. *et al.* The detection and estimation of linkage in polyploids using single-dose restriction fragments. *Theor. Appl. Genet.* **83**, 294–300 (1992).
2. Georganas, E. *et al.* HipMer: an extreme-scale de novo genome assembler. in *SC '15: Proceedings of the International Conference for High Performance Computing, Networking, Storage and Analysis* 1–11 (ieeexplore.ieee.org, 2015).
3. Vaser, R., Sović, I., Nagarajan, N. & Šikić, M. Fast and accurate de novo genome assembly from long uncorrected reads. *Genome Res.* **27**, 737–746 (2017).
4. Van Ooijen, J. W. JoinMap® 4, Software for the calculation of genetic linkage maps in experimental populations. *Kyazma BV, Wageningen*.
5. Torgo, L. *Data Mining with R, learning with case studies*. (Chapman and Hall/CRC, 2010).
6. Broman, K. W., Wu, H., Sen, S. & Churchill, G. A. R/qtl: QTL mapping in experimental crosses. *Bioinformatics* **19**, 889–890 (2003).
7. Monroe, J. G. *et al.* TSPmap, a tool making use of traveling salesperson problem solvers in the efficient and accurate construction of high-density genetic linkage maps. *BioData Min.* **10**, 38 (2017).
8. Cápal, P., Blavet, N., Vrána, J., Kubaláková, M. & Doležel, J. Multiple displacement amplification of the DNA from single flow–sorted plant chromosome. *Plant J.* **84**, 838–844 (2015).
9. Metcalfe, C. J. *et al.* Flow cytometric characterisation of the complex polyploid genome of Saccharum officinarum and modern sugarcane cultivars. *Sci. Rep.* **9**, 19362 (2019).
10. McCormick, R. F. *et al.* The Sorghum bicolor reference genome: improved assembly, gene annotations, a transcriptome atlas, and signatures of genome organization. *Plant J.* **93**, 338–354 (2018).
11. Li, H. & Durbin, R. Fast and accurate short read alignment with Burrows-Wheeler transform. *Bioinformatics* **25**, 1754–1760 (2009).

12. Quinlan, A. R. BEDTools: The Swiss-army tool for genome feature analysis. *Curr. Protoc. Bioinformatics* **47**, 11.12.1-34 (2014).

13. Koren, S. *et al.* Canu: scalable and accurate long-read assembly via adaptive k-mer weighting and repeat separation. *Genome Res.* **27**, 722–736 (2017).

14. Chin, C.-S. *et al.* Nonhybrid, finished microbial genome assemblies from long-read SMRT sequencing data. *Nat. Methods* **10**, 563–569 (2013).

15. Wang, M. & Kong, L. pblat: a multithread blat algorithm speeding up aligning sequences to genomes. *BMC Bioinformatics* **20**, 28 (2019).

16. Vollger, M. R. *et al.* Long-read sequence and assembly of segmental duplications. *Nat. Methods* **16**, 88–94 (2019).

17. Zhang, Z., Schwartz, S., Wagner, L. & Miller, W. A greedy algorithm for aligning DNA sequences. *J. Comput. Biol.* **7**, 203–214 (2000).

18. Dudchenko, O. *et al.* De novo assembly of the Aedes aegypti genome using Hi-C yields chromosome-length scaffolds. *Science* **356**, 92–95 (2017).

19. Durand, N. C. *et al.* Juicebox provides a visualization system for hi-C contact maps with unlimited zoom. *Cell Syst.* **3**, 99–101 (2016).

20. Marçais, G. *et al.* MUMmer4: A fast and versatile genome alignment system. *PLoS Comput. Biol.* **14**, e1005944 (2018).

21. Li, H. Minimap2: pairwise alignment for nucleotide sequences. *Bioinformatics* **34**, 3094–3100 (2018).

22. Zhang, J. *et al.* Allele-defined genome of the autopolyploid sugarcane Saccharum spontaneum L. *Nat. Genet.* **50**, 1565–1573 (2018).

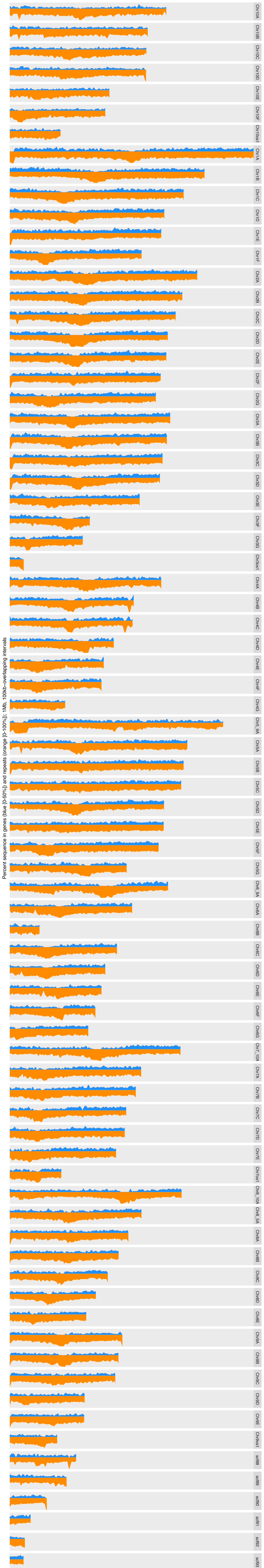

Supplementary Figure 12- R570 primary assembly gene and repeat densities. Each panel is separated into primary chromosomes and scaffolds with gene density (blue) shown on the top panel axis and repeat density (orange)shown on the bottom panel axis. Each density is represented as percent bases assigned per one megabasenon-overlapping window. The y-axis range for gene density is 0-50% (middle to top) and the y-axis range for repeat density is 0-100% (middle to bottom).

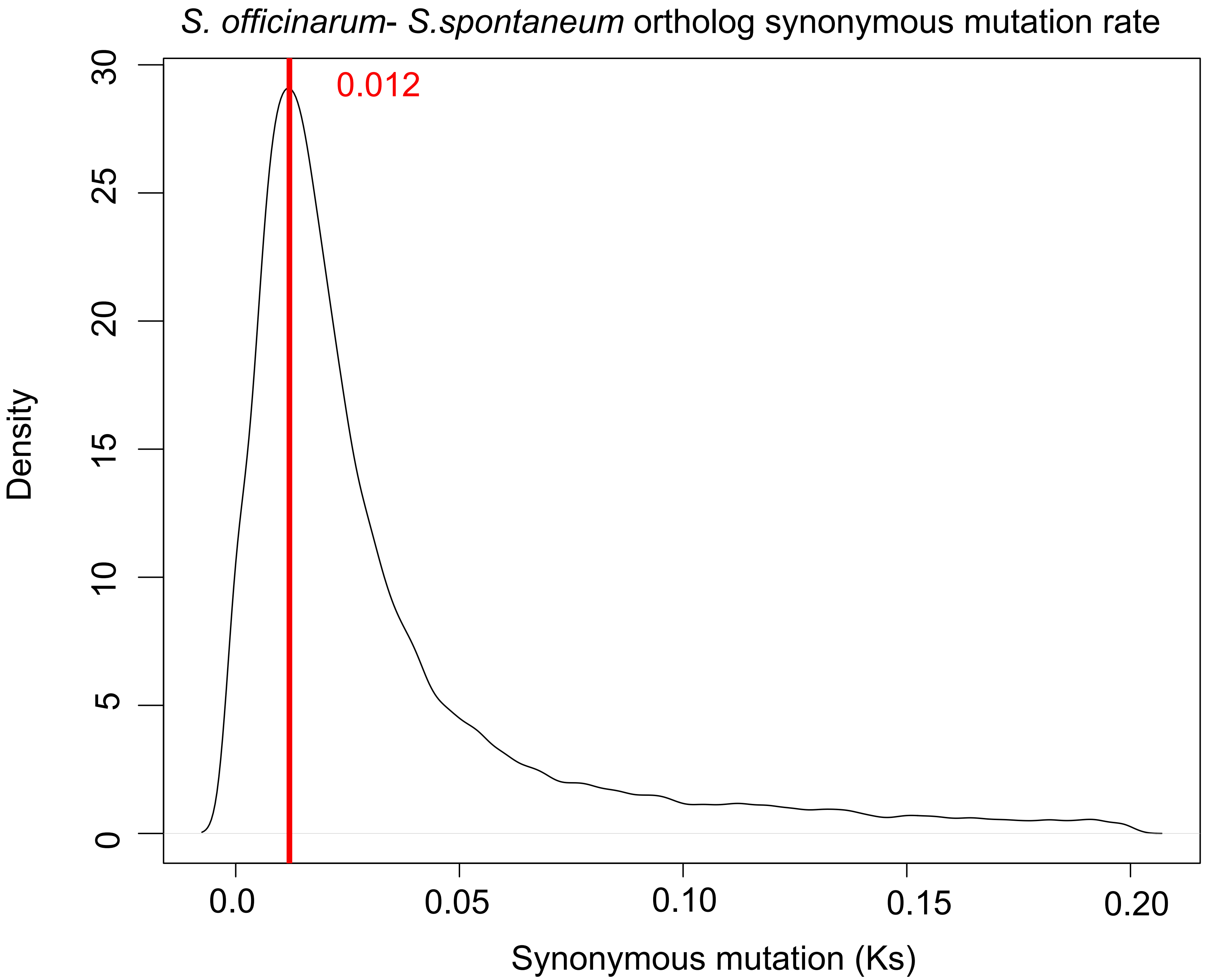

Supplementary Figure 13- Synonymous mutation peak among *S. officinarum* and *S. spontaneum* derived orthologs within cultivar R570. Forty five thousand random coding sequences (CDS) pairs were selected and compared. The synonymous mutation peak enabled inference of the divergence time between *S. officinarum* and *S. spontaneum* of approximately 1.55 million years.

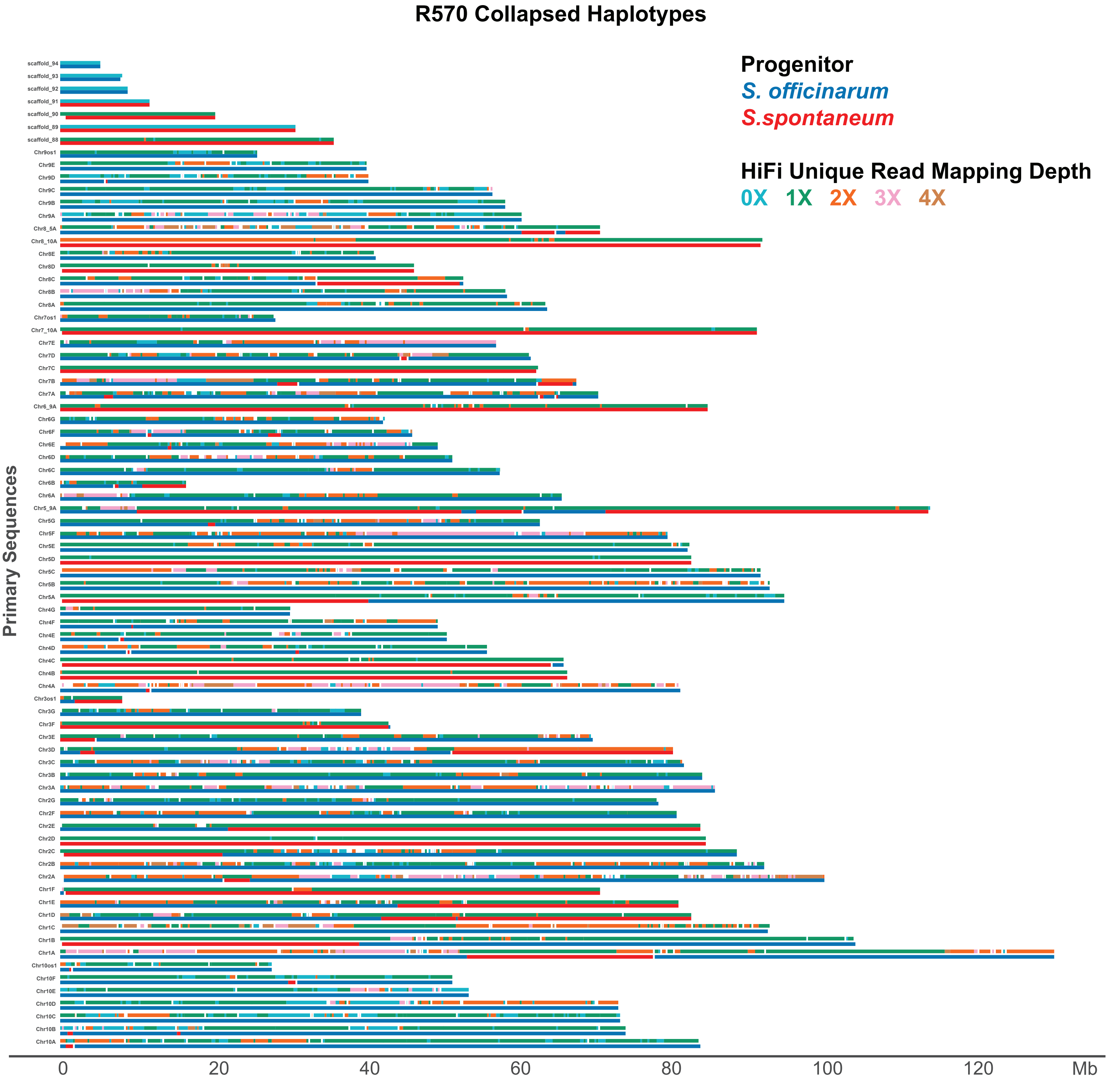

Supplementary Figure 14- Collapsed haplotypes within the R570 primary genome assembly. PacBio HiFi Reads were aligned to the R570 assembly and were used to calculate the amount of collapsed haplotypes across the genome. Regions with greater than 1X coverage represent multiple haplotypes that are collapsed due to perfectly homozygous, identical sequences. Example= 3x depth coverage = 1 represented haplotype + 2 collapsed haplotypes. 0X unique read mappings are localized regions of the genome where the HiFiAsm assembler (utilizing sequence overlap) was to separate and assemble contigs representing nearly identical haplotypes, but individual HiFi reads independently aligned cannot distinguish between them, resulting in zero uniquely mapping reads. Most often, the other identical haplotype is located in the alternate assembly.

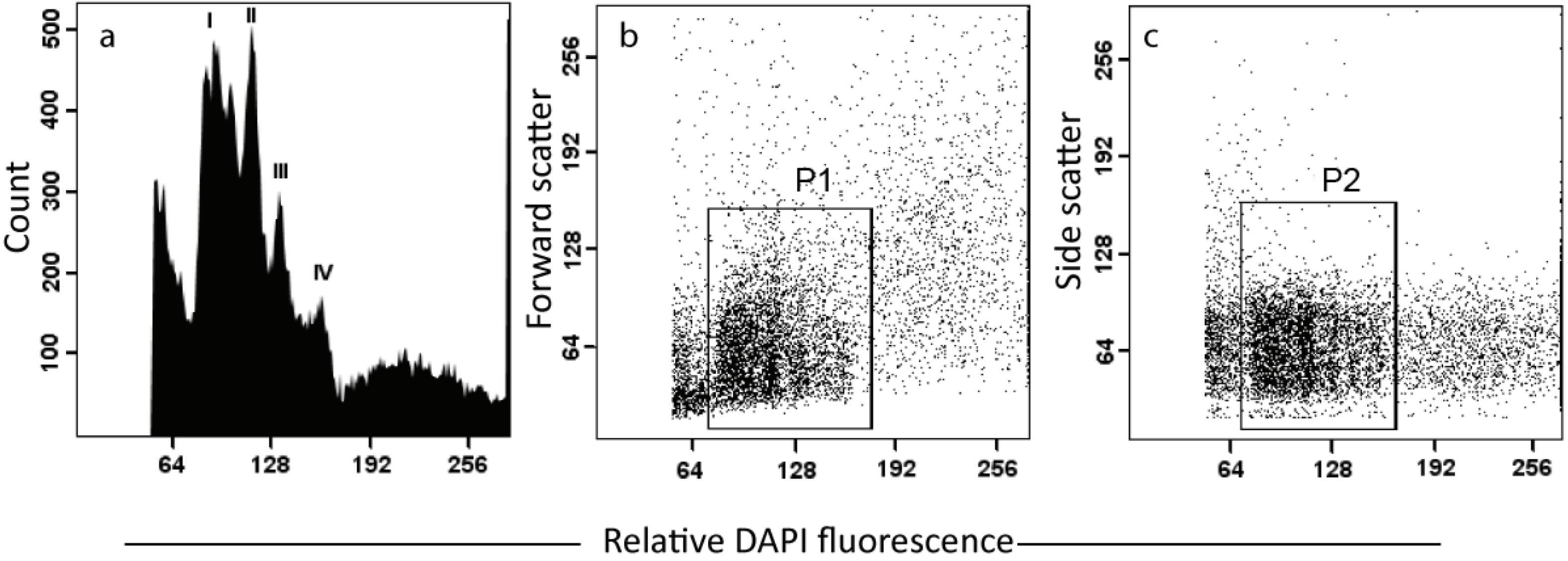

Supplementary Figure 15- Flow sorting of sugarcane mitotic chromosomes. a, Histogram of chromosome fluorescence intensity (flow karyotype) of sugarcane cultivar R570. b, Scatter plot of relative DAPI fluorescence vs. forward scatter parameter. Initial gate P1 was drawn around the chromosomal population. c, Dependent final sorting gate P2 was drawn in biparametric scatter plot relative DAPI fluorescence-area vs. relative DAPI fluorescence-width to exclude chromosome doublets.
