# Supplementary material for: The complex polyploid genome architecture of sugarcane
Source: Nature. 2024 Mar 27;628(8009):804–10. doi: 10.1038/s41586-024-07231-4 (PMC11041754; doi:10.1038/s41586-024-07231-4)
Supplement: Supplementary file 2 — Reporting Summary [file 41586_2024_7231_MOESM2_ESM.pdf]

nature portfolio

Corresponding author(s): Adam Healey

Last updated by author(s): Feb 13, 2024

Reporting Summary

Nature Portfolio wishes to improve the reproducibility of the work that we publish. This form provides structure for consistency and transparency in reporting. For further information on Nature Portfolio policies, see our Editorial Policies and the Editorial Policy Checklist.

Statistics

For all statistical analyses, confirm that the following items are present in the figure legend, table legend, main text, or Methods section.

n/a	Confirmed	
☐	☒	The exact sample size (n) for each experimental group/condition, given as a discrete number and unit of measurement
☒	☐	A statement on whether measurements were taken from distinct samples or whether the same sample was measured repeatedly
☐	☒	The statistical test(s) used AND whether they are one- or two-sided<br>*Only common tests should be described solely by name; describe more complex techniques in the Methods section.*
☒	☐	A description of all covariates tested
☒	☐	A description of any assumptions or corrections, such as tests of normality and adjustment for multiple comparisons
☐	☒	A full description of the statistical parameters including central tendency (e.g. means) or other basic estimates (e.g. regression coefficient) AND variation (e.g. standard deviation) or associated estimates of uncertainty (e.g. confidence intervals)
☒	☐	For null hypothesis testing, the test statistic (e.g. F, t, r) with confidence intervals, effect sizes, degrees of freedom and P value noted<br>*Give P values as exact values whenever suitable.*
☒	☐	For Bayesian analysis, information on the choice of priors and Markov chain Monte Carlo settings
☒	☐	For hierarchical and complex designs, identification of the appropriate level for tests and full reporting of outcomes
☒	☐	Estimates of effect sizes (e.g. Cohen's d, Pearson's r), indicating how they were calculated

Our web collection on statistics for biologists contains articles on many of the points above.

Software and code

Policy information about availability of computer code

Data collection	No software was used for data collection for this manuscript.
Data analysis	Genome Assembly: RACON(v1.4.10), HiFiAsm (v0.13-r308), Juicebox (v 1.11.08)<br>Genetic Map Construction: BWA-MEM (v0.7.12), JoinMap (v4.0), R/QTL (v1.42-8), DMwR (v0.4.1)<br>Optical Map Construction: Bionano tools (v1.3.8041.8044), Bionano Solve (v3.3_10252018), Bionano Genomics Access software (Tools 1.3)<br>Genome Assembly: pblat (v2.5) GENESPACE (v0.9.4), minimap2 (v 2.20-r1061)<br>Genome Annotation: GSNAP (v2013-09-30); PASA (v2.0.2);EXONERATE (v2.4.0) ; RepeatModeler (v.open1.0.11); FGENESH+(v3.1.0); AUGUSTUS (v3.1.0)<br>Comparative Genomics: GENESPACE (v0.9.4), Orthofinder (v2.5.4), MCScanX (v2), SyRI (v1.6), Biostrings (v2.70.2), MAFFT (v7.487), seqinr (v4.2-16), ESMfold (v2.0.1), MAFFT (v7.487), NLR-Annotator (v2), pal2nal (v13), Olympus Cellsens software (v), FACSDiva (v6.1.3), Summit (v 6.2.2),Olympus Cellsens software (v3.2)<br>Other custom data scripts and raw data files required for analysis have been provided and uploaded to FigShare (https://doi.org/10.6084/m9.figshare.22138004) or Github (https://github.com/a-healey/r570scripts) for free use.

For manuscripts utilizing custom algorithms or software that are central to the research but not yet described in published literature, software must be made available to editors and reviewers. We strongly encourage code deposition in a community repository (e.g. GitHub). See the Nature Portfolio guidelines for submitting code & software for further information.

Data

Policy information about availability of data

All manuscripts must include a data availability statement. This statement should provide the following information, where applicable:

- Accession codes, unique identifiers, or web links for publicly available datasets
- A description of any restrictions on data availability
- For clinical datasets or third party data, please ensure that the statement adheres to our policy

Sequencing libraries (Illumina DNA/RNA and PacBio CLR/HiFi) are publicly available within the sequence read archive (SRA). BioProjects and individual accession numbers are provided in Supplemental Table 14. Genome assembly and annotation for the primary are freely available at Phytozome (https://phytozome-next.jgi.doe.gov/). This Whole Genome Shotgun project has been deposited at DDBJ/ENA/GenBank under the accession JAQSUU000000000. The version described in this paper is version JAQSUU010000000. Publicly available genomes used for comparative genomics can be downloaded here: Setaria viridis (v2.1; https://phytozome-next.jgi.doe.gov/info/Sviridis_v2_1), Sorghum bicolor (v3.1; https://phytozome-next.jgi.doe.gov/info/Sbicolor_v3_1_1), R570 monoploid tiling path (http://sugarcane-genome.cirad.fr), Saccharum spontaneum (http://www.life.illinois.edu/ming/downloads/Spontaneum_genome/). Raw data used for analysis in this paper are freely available on figshare (https://doi.org/10.6084/m9.figshare.22138004).

Research involving human participants, their data, or biological material

Policy information about studies with human participants or human data. See also policy information about sex, gender (identity/presentation), and sexual orientation and race, ethnicity and racism.

Reporting on sex and gender	Not applicable
Reporting on race, ethnicity, or other socially relevant groupings	Not applicable
Population characteristics	Not applicable
Recruitment	Not applicable
Ethics oversight	Not applicable

Note that full information on the approval of the study protocol must also be provided in the manuscript.

Field-specific reporting

Please select the one below that is the best fit for your research. If you are not sure, read the appropriate sections before making your selection.

☒ Life sciences ☐ Behavioural & social sciences ☐ Ecological, evolutionary & environmental sciences

For a reference copy of the document with all sections, see nature.com/documents/nr-reporting-summary-flat.pdf

Life sciences study design

All studies must disclose on these points even when the disclosure is negative.

Sample size	Sample size was not predetermined prior to analysis. Selfed offspring genotyped for the genetic map (n=96) we selected based on budgetary constraints.
Data exclusions	No data was excluded from analyses.
Replication	DNA/RNA was extracted from verified clones of the same genotype (R570). For repeatability, the same clone (held in greenhouses) could be selected and tested.
Randomization	Genome assembly and analysis were conducted on a single sugarcane hybrid genotype 'R570', thus randomization is not necessary.
Blinding	Genome assembly and analysis were conducted on a single sugarcane hybrid genotype 'R570', thus blinding is not necessary.

Reporting for specific materials, systems and methods

We require information from authors about some types of materials, experimental systems and methods used in many studies. Here, indicate whether each material, system or method listed is relevant to your study. If you are not sure if a list item applies to your research, read the appropriate section before selecting a response.

Materials & experimental systems

n/a	Involved in the study	
☒	☐	Antibodies
☒	☐	Eukaryotic cell lines
☒	☐	Palaeontology and archaeology
☒	☐	Animals and other organisms
☒	☐	Clinical data
☒	☐	Dual use research of concern
☐	☒	Plants

Methods

n/a	Involved in the study	
☒	☐	ChIP-seq
☐	☒	Flow cytometry
☒	☐	MRI-based neuroimaging

Dual use research of concern

Policy information about dual use research of concern

Hazards

Could the accidental, deliberate or reckless misuse of agents or technologies generated in the work, or the application of information presented in the manuscript, pose a threat to:

No	Yes	
☒	☐	Public health
☒	☐	National security
☒	☐	Crops and/or livestock
☒	☐	Ecosystems
☒	☐	Any other significant area

Experiments of concern

Does the work involve any of these experiments of concern:

No	Yes	
☒	☐	Demonstrate how to render a vaccine ineffective
☒	☐	Confer resistance to therapeutically useful antibiotics or antiviral agents
☒	☐	Enhance the virulence of a pathogen or render a nonpathogen virulent
☒	☐	Increase transmissibility of a pathogen
☒	☐	Alter the host range of a pathogen
☒	☐	Enable evasion of diagnostic/detection modalities
☒	☐	Enable the weaponization of a biological agent or toxin
☒	☐	Any other potentially harmful combination of experiments and agents

Plants

Seed stocks	Plant material used for sequenced was derived from sugarcane hybrid cultivar R570, held in greenhouses at CIRAD research station in Montpellier, France
Novel plant genotypes	No novel genotypes used in this study.
Authentication	No authentication procedures required.

Flow Cytometry

Plots

Confirm that:

☒ The axis labels state the marker and fluorochrome used (e.g. CD4-FITC).

☒ The axis scales are clearly visible. Include numbers along axes only for bottom left plot of group (a 'group' is an analysis of identical markers).

☒ All plots are contour plots with outliers or pseudocolor plots.

☐ A numerical value for number of cells or percentage (with statistics) is provided.

Methodology

Sample preparation	Roots for single chromosome sorting were harvested from R570 plants, grown in pots. Roots were treated with 0.04% hydroxyquinoline for 3 h, fixed for 72 h in 3 : 1 methanol: acetic acid solution and stored in 75% ethanol at 4 degrees C. The fixed roots were rinsed twice in water for 10 min each, treated in 0.25 N HCl for 10 min, rinsed for 10 min in water and placed in digestion buffer (0,01 M citrate buffer pH 4.5, 0.075 M KC1) for 10 min. The root tips were cut and placed in an enzyme solution (5% Onozuka R-10 cellulase, 1% Y-23 pectolyase in digestion buffer) in a microtube at 37 ° C for approximately 3 h (the time varying with the size of the roots), Root tips were then rinsed in water and spread on a slide with a drop of 3:1 ethanol:acetic acid.- This strategy for staining sugarcane chromosomes was first described in D'hont et al. 1996 "Characterisation of the double genome structure of modern sugarcane cultivars (Saccharum spp.) by molecular cytogenetics"
Instrument	Images were captured with a CCD camera attached to a BX53 Olympus microscope.
Software	Olympus Cellsens software (v3.2)
Cell population abundance	Individual cells were not used, rather chromosomes that were arrested in metaphase and stained. Thus, a numerical value for cells is not relevant.
Gating strategy	Relative fluorescence intensity was used to separate flow karyotype peaks (I-V) and isolate single chromosomes for sequencing.

☒ Tick this box to confirm that a figure exemplifying the gating strategy is provided in the Supplementary Information.
